# Enhanced Photovoltaic Performance of Asymmetrical Benzo Dithiophene Homopolymer Donor Materials in Nonfullerene Acceptor-Based Organic Photovoltaics

**DOI:** 10.3390/molecules29061332

**Published:** 2024-03-17

**Authors:** Wei Xu, Li Du, Zhengkun Du, Wei He, Hongxiang Li, Guojuan Li, Cheng Yang, Pei Cheng, Zhong Cao, Donghong Yu

**Affiliations:** 1 Hunan Provincial Key Laboratory of Materials Protection for Electric Power and Transportation, Hunan Provincial Key Laboratory of Cytochemistry, School of Chemistry and Chemical Engineering, Changsha University of Science and Technology, Changsha 410114, China; xw654751046@163.com; 2 Department of Chemistry and Bioscience, Aalborg University, Fredrik Bajers Vej 7H, DK-9220 Aalborg, Denmark; 3 College of Energy Storage Technology, Shandong University of Science and Technology, Qingdao 266590, China; dl632195374@163.com; 4 State Key Laboratory of Polymer Materials Engineering, Department of College of Polymer Science and Engineering, Sichuan University, Chengdu 610065, China; hewei1999scu@163.com (W.H.); lihongxiang@scu.edu.cn (H.L.); chengpei@scu.edu.cn (P.C.); 5 National Anti-Drug Laboratory Sichuan Regional Center, Chengdu 610206, China; liguojuan1230@163.com; 6 Key Laboratory of Green Chemistry and Technology, State Key Laboratory of Biotherapy, College of Chemistry, Sichuan University, Chengdu 610064, China; yangchengyc@scu.edu.cn; 7 Sino-Danish Center for Education and Research, DK-8000 Aarhus, Denmark

**Keywords:** benzodithiophene, donor polymer, photovoltaic, organic solar cells, power conversion efficiency

## Abstract

Although much promising synthetic progress in conjugated polymer-based organic solar cells (OSCs) has resulted in significant improvement in power conversion efficiencies (PCEs) of from over 15 to >19.0% in the last five years, the sophisticated and complex reactions from at least two families’ monomers with remarkably different electron push–pull effects could still pose an unavoidable material burden for the commercialization of OSCs in the coming future. Therefore, the method of preparing a homopolymer from a sole monomer would significantly reduce the synthetic steps and costs in order to pave the way for the large-scale production of OSC materials. Therefore, alkylthio-thiophenyl-substituted benzo[1,2-b;4,5-b′]dithiophene (BDTTS) as the sole and key structural moiety with dihalogen and distannyl functional groups was designed and synthesized, respectively, in this study, for facile monomer syntheses and polymerizations to achieve three wide-bandgap homopolymer donors of BDTTS-*alt*-BDTT-Cl (P13), BDTTS-*alt*-BDTT (P15), and BDTTS (P14), respectively. The structural symmetry dependency on their physical, electrochemical, and optical properties, thin-film morphologies, and photovoltaic (PV) performance was investigated in detail. As a result, OSCs based on the asymmetric polymer P15, paired with BTP-eC9 as the electron acceptor, presented the best PV performance, with a PCE of 11.5%, a fill factor (FF) of 65.87%, and a short-circuit current (*J*_SC_) of 22.04 mA·cm^−2^, respectively. This PCE value is among the highest ones reported for BDT-type homopolymer donor-based OPVs, providing us with knowledge for obtaining promising PV performance from devices made of P15-like materials.

## 1. Introduction

The structural design of polymer donor materials plays an important role in improving the energy conversion efficiency of polymer solar cells (PSCs). Recently, copolymers with electron push–pull effects from electron-donor (D) and electron-acceptor (A) alternating units have attracted considerable attention as potential donor materials in PSCs [1,2,3]. However, these D–A copolymers naturally demand multiple synthetic steps to produce two or more different types of monomers, which costs more time and raw materials [4,5]. Although the prices of popular polymer donor materials are affordable for scientific studies in laboratories, their costs remain high for commercialization. In order to solve this dilemma, homopolymers, derived from only one type of key monomer moiety, could serve as an ideal alternative to D–A copolymers that use a second type of electron-acceptor monomer, being both resource- and cost-effective via significantly shortened synthetic pathways. Actually, investigations into homopolymers had already commenced over a decade ago, and the most successful one was poly(3-hexylthiophene) (P3HT) till now [6,7,8]. Nonetheless, the energy level limitations of its molecular orbitals and the difficulties of the structural modifications to P3HT restricted its further development. Because P3HT has a narrow absorption in the visible region with no cross-over with its state-of-the-art fullerene derivative acceptors, it has a weak light-harvesting capability. Furthermore, its relatively higher HOMO energy level means that P3HT-based PSC devices usually have lower short current densities (*J*_SC_) and open circuit voltages (V_OC_). Thus, such homopolymers with novel electron-donating units are expected to overcome this predicament. 

So far, extensive efforts have been made to find an alternative homopolymer with the advantages of a simple structure and high performance. In 2015, Kim B.J. and his colleagues reported a potential homopolymer based on alkyl-thienyl benzodithiophene (BDT) units (PBDTT), which obtained a nice PCE of 6.1% in its PC_71_BM-based PSCs [9]. Compared with P3HT, BDT-based homopolymers have a broader optical bandgap, a higher absorption coefficient, lower HOMO levels, and better thermal stability. Then, besides the novel BDT-based homopolymer with alkylthio-thienyl side chains, Hwang et al. also first reported an asymmetrical BDT-based homopolymer with different flanks [10]. They found that devices from an alkylthio sidechain-substituted BDT-based homopolymer (PBDTT-S) performed with a higher PCE of 7.05%, a higher V_OC_ of 0.99 V, and a better *J*_SC_ of 13.92 mA·cm^−2^ than PBDTT-based ones. And the asymmetrical homopolymer (PBDTT-BDTT-S) exhibited moderate photovoltaic (PV) performance among three homopolymers in fullerene-based PSCs. Subsequently, Wong and co-workers synthesized a fluorine flank-substituted asymmetrical BDT-based homopolymer (PBBF) and applied it into bis(dicycanovinylindan-1-one (IC))-capped indacenodithiophen (IDT) core molecule (IDIC)-based PSCs to achieve a higher PCE of 8.5% [11]. Obviously, the PV performance of the PBBF-based PSCs was better than that of the PBDTT-based ones in all aspects. In addition to the optical absorption enhancement and HOMO level deepening brought about by fluorination, which simultaneously increased *J*_SC_ and V_OC_, the asymmetrical homopolymer also had better film morphology, inducing improved fill factors (FFs). These results demonstrated that one future bright structural design idea would be asymmetrical homopolymer donor materials that could achieve better PV performance. And nowadays, there are not sufficient reports found on studies of the syntheses and PV properties of such asymmetric homopolymers. Accordingly, further efforts should be aimed at the following: (1) deepening the HOMO energy level with a trade-off between V_OC_ and *J*_SC_ and (2) increasing the light absorption coefficient and improving the generation and mobility of charges to maximize *J*_SC_ and FFs.

In this study, we designed and synthesized two BDT-based asymmetric homopolymers with properly selected flanks via Stille coupling reactions, i.e., poly{4,8-bis(5-((2-ethylhexyl)thio)thiophen-2-yl)-benzo[1,2-b:4,5-b′]dithiophene-alt-4,8-bis(4-chloro-5-(2-ethylhexyl))thiophen-2-yl)-benzo[1,2-b:4,5-b′]-dithiophene} (P13 or PBDTT-S-BDTT-Cl) and poly{4,8-bis(5-((2-ethylhexyl)thio)thiophen-2-yl)-benzo[1,2-b:4,5-b′]dithiophene-alt-4,8-bis(5-(2-ethylhexyl))thiophen-2-yl)-benzo[1,2-b:4,5-b′]dithiophene} (P15 or PBDTT-BDTT-S). Moreover, we also prepared a contrastive homopolymer, poly{4,8-bis(5-((2-ethylhexyl)thio)thiophen-2-yl)benzo[1,2-b:4,5-b′]-dithiophene} (P14 or PBDTT-S), to highlight the advantages of asymmetric ones. Interestingly, the asymmetric homopolymers (P13 and P15) exhibited better PV performance than the symmetric homopolymer (P14), among which P15 showed notably improved charge dissociation, charge recombination, and hole mobility, affording a promising PCE of 11.5%, which is the highest value reported for devices from BDT-based donor-1–donor-2 (D1-D2)-type homopolymers.

## 2. Results and Discussion

**Figure 1:**
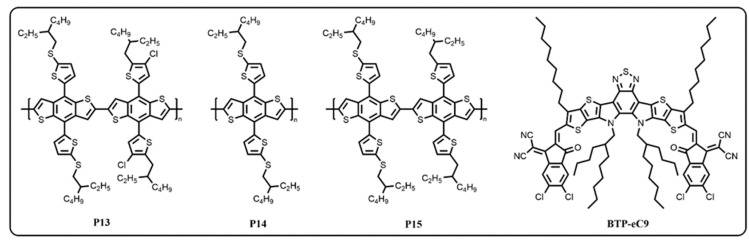
Schematic diagram of the molecular structures of synthetic BDT-based D1-D2-type homopolymers and the acceptor of BTP-eC9 paired in the active layer of the OSC devices.

The chemical structures of BDT-based D1-D2-type homopolymers are shown in Figure 1, and their synthetic pathways are outlined in Scheme S1 of the Supplemental Electric Information (ESI), which happen via copolymerizing with 4,8-bis(5-((2-ethylhexyl)thio)thiophen-2-yl)benzo[1,2-b:4,5-b′]-dithiophene (BDTT-S), 4,8-bis(4-chloro-5-(2-ethylhexyl))thiophen-2-yl)benzo[1,2-b:4,5-b′]-dithiophene (BDTT-Cl), or 4,8-bis(5-(2-ethylhexyl))thiophen-2-yl)benzo[1,2-b:4,5-b′]-dithiophene (BDTT) monomers using Pd_2_(dba)_3_ as a catalyst. Furthermore, the crude homopolymers were further purified successively with methanol, acetone, *n*-hexane, and chloroform by means of Soxhlet extraction. The homopolymers were readily dissolved in most organic solvents, such as chloroform (CF), chlorobenzene (CB), and *o*-dichlorobenzene (*o*-DCB), which indicated that they are all easily solution-processable [12]. Through gel permeation chromatography (GPC), the number-average molecular weight (Mn) and the polydispersity index (PDI) of the homopolymers were measured with calibration against polystyrene standards and were found to be 3.43 kDa with a PDI of 2.58 for P13, 2.89 kDa with a PDI of 2.20 for P14, and 2.79 kDa with a PDI of 2.16 for P15, respectively. As the organic semiconductor materials have state-of-the-art conjugated backbone structures (Figure S1), all the polymer materials were thermally stable at their decomposition temperatures (as the temperature that causes their 5% weight loss) of 348 °C for P13, 315 °C for P14, and 341 °C for P15, respectively.

**Figure 2:**
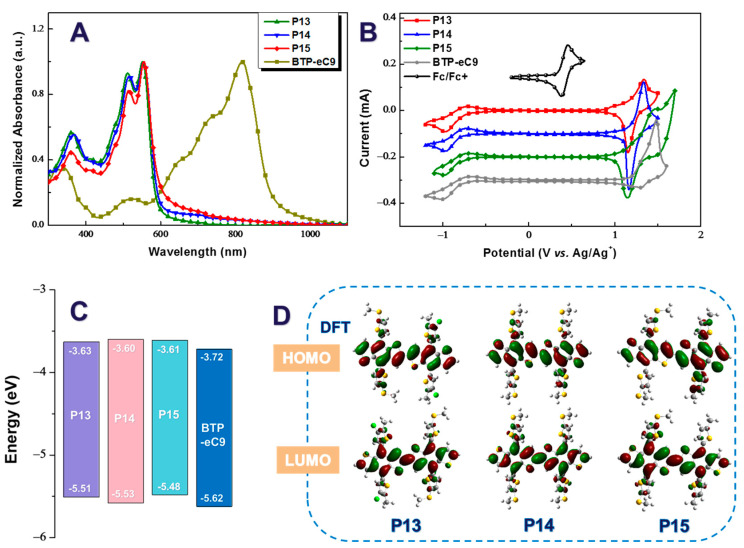
UV-vis absorption spectra (**A**), CV charts (**B**), molecular energy levels (**C**), and the calculated electron distributions of the HOMO/LUMO orbitals (**D**) of the polymers P13, P14, and P15 and the acceptor BTP-eC9.

Then, their optical properties were characterized by means of ultraviolet–visible spectroscopy, as shown in Figure 2A. The UV-vis absorption spectra of the three polymers in thin films indicated that P15 exhibited the best optical properties with the broadest absorption edge. And the optical absorption edges of these homopolymers were found to be 630, 635, and 640 nm for P13, P14, and P15, respectively, which corresponded to optical bandgaps of 1.97, 1.95, and 1.94 eV for P13, P14, and P15, respectively, with good complementary absorbance to that of the acceptor BTP-eC9 in the visible light region. Moreover, the thin-film electrochemical characteristics of the three polymers were studied by using cyclic voltammetry (CV) (Figure 2B), and their onset oxidation potentials (E_OX_) were found to be 1.13, 1.15, and 1.10 V for P13, P14, and P15, respectively, corresponding to HOMO energy levels of −5.51, −5.53, and −5.48 eV for P13, P14, and P15, respectively. The HOMO level of P14 was undoubtably the lowest one among them, which could be attributed to the strongest electron-withdrawing effect of alkylthio sidechains. And the LUMO energy levels of these homopolymers were obtained by the same means as exhibited in Table 1. Thus, the electronic bandgaps (E_g_) of the three polymers could be calculated, which approximated their optical bandgaps, E_g_^opt^. Via optimization and calculation by means of density functional theory (DFT), energy level schematic diagrams of these homopolymers are presented in Figure 2D. It can clearly be seen that the electron clouds in the HOMO energy levels of P13 and P14 were almost evenly distributed along the polymer backbone, while that of P15 was favorably centered in the BDTT moieties. The optimized results revealed that the electron push–pull effect of P15 was significant between the BDTT-S and BDTT units, caused by the difference in the inductive effect between alkyl-thienyl sidechains and alkylthio-thienyl sidechains, which is similar to results for D–A alternating copolymers.

To further investigate the thin-film properties of the organic active layers based on the three homopolymers, the DFT method was used to simulate their optimal thermodynamic structures [12,13]. The dihedral angles between the planes of the focused structural moieties in their polymer backbones were calculated and are presented in Figure S2. These homopolymers had similar dihedral angles between their thienyl sidechains and BDT core. However, P14 exhibited the minimum dihedral angle between two adjacent BDT units, which was attributed to the symmetrical structure of the polymer backbone. And another result also proved the above inference: P13 substituted with chloride thienyl sidechains had the largest dihedral angle due to its mostly asymmetrical structure. As far as the theoretical results are concerned, the smaller dihedral angles corresponded to flatter conjugated backbones, which could indicate that P14 and P15 would form closer π–π stacking than P13 did in thin films.

To study the PV performance of these homopolymer donor materials, we applied them to practical OPV devices with conventional structures, as described above in the fabrication of OPV devices in the Materials and Methods section. Through many optimizations of the blending weight ratio between the donors and acceptors, we confirmed an optimal blending weight ratio of 1:1 (*wt*/*wt*) for the active layer based on these homopolymers. And on the basis of such an optimal weight ratio, we carried out further optimizations by adjusting the content of the solvent additive 1,8-diiodooctane (DIO). The optimization processes for the content of this solvent additive in active layers based on these three homopolymers are presented in Figure S3 and the table included. Both P13 and P15 had the same optimal content of solvent additive of 0.1% (volume percentage), and the P14-based OPV devices performed optimally under the pristine blending condition, which means that P14-based OPV devices were strongly negatively sensitive to DIO. For P13, when the content of the solvent additive in the active layer increased from 0 to 0.1%, the FF presented an increase of 7.9%, *J*_SC_ underwent a decrease of 4.8%, and V_OC_ remained unchanged. An increase from 0 to 0.3% in DIO led to an increase of 10.0% for the FF but a decrease of 13.9% for *J*_SC_, which indicated that DIO had a moderate effect on the P13-based active layers. For P15, when the content of the additive increased from 0 to 0.1%, the FF underwent an increase of 6.8%, *J*_SC_ underwent an increase of 0.3%, and V_OC_ underwent an increase of 1.3%. When the DIO content increased from 0 to 0.3%, the FF revealed an increase of 4.5%, *J*_SC_ underwent an increase of 0.6%, and V_OC_ maintained an increase of 1.3%. These results indicate that DIO only exhibited a positive effect on the P15-based active layer. 

**Figure 3:**
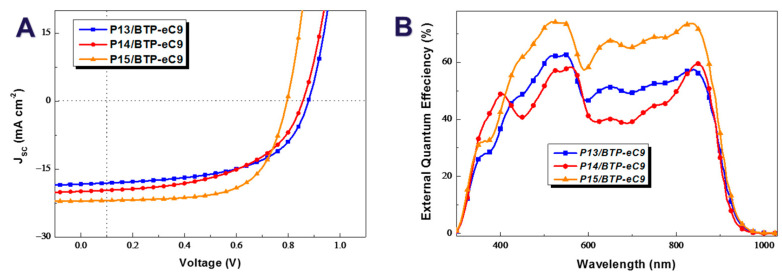
J–V curves (**A**) and EQE plots (**B**) of the optimal OPV devices based on the three homopolymers P13, P14, and P15.

Hence, we obtained optimal PV performance for OPV devices based on these homopolymers. As shown in both Figure 3A and Table 2, although their V_OC_ was the lowest among the three, the P15-based OPV devices performed the best, with a PCE of 11.53% (with an average PCE of 11.08%, obtained from 10 parallel devices), the highest FF of 65.87%, and the highest *J*_SC_ of 22.04 mA·cm^−2^. Remarkably, this is the highest value reported for devices based on such donor-1 (D1)–donor-2 (D2)-type wide-bandgap homopolymer donors with regard to their PV performance, as seen in the overview in Table S1 and the references therein, which is caused by the structural contribution of the asymmetry between the alkylated and alkylthiolated thiophenyl BDTs. There was no doubt that the lowest V_OC_ for the P15-based devices resulted from it having the highest HOMO level among the three homopolymers. Compared with P14, the P13-based OPV devices had a higher FF of 57.1% and V_OC_ of 0.88 V, resulting in their higher PCE of 9.18% (Ave. 8.63%) than that of 9.07% (Ave. 8.49%) for the P14-based ones, owing to their having the lowest FF, although they had moderate values for *J*_SC_ and V_OC_. According to external quantum efficiency (EQE) plots (Figure 3B), the integrated *J*_SC_ of the optimal homopolymer-based OPV devices were calculated as 17.68, 19.33, and 21.44 mA·cm^−2^ for P13, P14, and P15, respectively, which were consistent with their J–V experimental results. Interestingly, P14 had the lowest HOMO energy level, but its corresponding OPV devices did not exhibit the highest V_OC_ due to their higher voltage loss. Overall, all three homopolymers pronounced larger *J*_SC_ values than other homopolymers reported and also achieved the best PCE [9,10,11]. In particular, the P15-based OPV devices had an outstanding PCE of 11.53%, which surpassed the formerly most efficient homopolymer-based OPV devices with a 20% improvement in PCE [11]. This can be clearly seen in Table S1 and the reference therein. In general, when coupling with BTP-eC9, the OPV devices based on these three D1-D2-type homopolymer donors, P13, P14, and P15, all had excellent PV performance. 

To further investigate the working mechanism of the three homopolymer material-based PSCs, their semiconductor characteristics (Table 3) were characterized following the instructions described in the Materials and Methods Section. The hole mobilities of homopolymer-based PSCs without DIO were found to be 5.03 × 10^−5^, 1.26 × 10^−5^, and 4.88 × 10^−5^ cm^2^ V^−1^ s^−1^ for P13, P14, and P15, respectively. After adding the additive DIO, the hole mobilities were found to be 3.65 × 10^−5^, 7.86 × 10^−6^, and 2.30 × 10^−4^ cm^2^ V^−1^ s^−1^ for P13, P14, and P15, respectively. And the P_diss_ (see Figure S6 in ESI for details) of the P13-, P14-, and P15-based OPVs (with or without DIO) are listed in Table 3. In addition, charge recombination was characterized by function diagrams of the light intensity against *J*_SC_ and V_OC_ in the OPVs (details found in Figure S5). All the homopolymers had excellent P_diss_ values in the range of 0.97~0.99 in their corresponding PSC devices with or without DIO, which indicated that bimolecular recombination could almost completely be ignored. But it was notable that the slopes of V_OC_ ∝ ln(I), denoted as n·kT/q, decreased when adding the DIO additive, which indicated that the DIO additive induced reductions in the trap-assisted recombination inside the homopolymer-based OPV devices. Obviously, the optimal PSC devices based on P15 performed the best in terms of hole mobility, charge dissociation ability, and having the weakest charge recombination among the three. 

**Figure 4:**
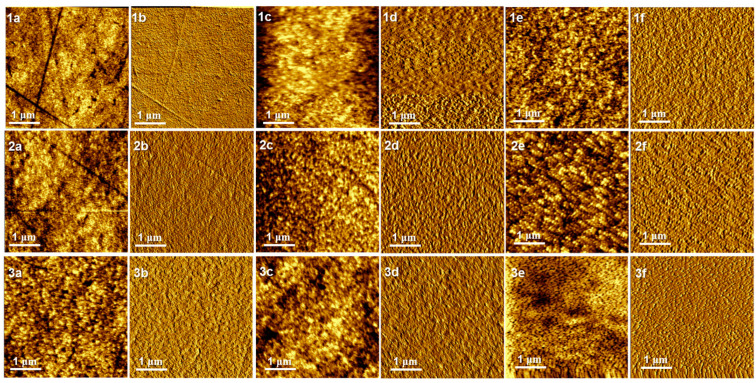
AFM height and phase diagrams of the thin films of pristine P13 (height **1a**, phase **1b**), pristine P14 (height **2a**, phase **2b**), pristine P15 (height **3a**, phase **3b**), P13:BTP-eC9 (height **1c**, phase **1d**), P14:BTP-eC9 (height **2c**, phase **2d**), P15:BTP-eC9 (height **3c**, phase **3d**), P13:BTP-eC9 with 0.1% DIO (height **1e**, phase **1f**), P14:BTP-eC9 with 0.1% DIO (height **2e**, phase **2f**), and P15:BTP-eC9 with 0.1% DIO (height **3e**, phase **3f**); 4 µm × 4 µm.

Since the actual morphology of these thin films plays an important role in charge separation at D–A interfaces and charge transport in the D and A domains, to determine the FF values [14,15], investigations by means of atomic force microscopy (AFM) and grazing-incidence wide-angle X-ray scattering (GIWAX) were carried out in depth. Therefore, we first characterized the morphologies of the three homopolymer thin films and their corresponding thin films of D/A blends by using AFM (see Figure 4). Generally, the surface morphologies of these thin films presented less roughness, uniform particles and good miscibility.

It is shown, as seen in Figure 4(1a–3a,1b–3b), that all the pristine homopolymer thin films, P13, P14, and P15, respectively, showed oriented and parallel thin lines like fibers in their phase diagrams, which demonstrated that all the homopolymer chains were ordered and in close arrangement due to π–π stacking along their polymer backbones. One can see in 1c–3c (height) and 1d–3d (phase) in Figure 4 for the films of the P13/, P14/, and P15/BTP-eC9 blends that their granule sizes in thin films became larger. All the oriented and parallel arrangements of polymer backbones turned into a well-developed nanoscale bicontinuous interpenetrating network with a fibrous structure. We attributed this to the strong interactions with the acceptor BTP-eC9, which enhanced the crystallization of the polymer/BTP-eC9 particles and stimulated a dense π–π stacking interaction between the donors and acceptors [16,17,18]. Furthermore, according to the height and phase diagrams of the blended thin films (Figure 4(1c–3c,1d–3d)), the three homopolymers (again, see Figure 4(1a–3a,1b–3b)) all exhibited excellent surface phase separation, which was beneficial for charge separation and recombination. Obviously, the blended thin films of P13 and P14 were both changed from a densely arranged morphology to a loose and porous morphology, although they formed larger crystalline particles. This indicated an intense aggregation effect and strong crystallinity, resulting in unfavorable factors such as excessive phase separation and unbalanced charge transport for the corresponding OPV devices [19,20,21,22]. Thus, these blended OPV devices with the DIO additive (whose morphologies follow Figure 4 (1e–3e, 1f–3f)) had a higher FF and a lower *J*_SC_ compared with the pristine blend-based OPVs. On the other hand, the OPV devices from P15 present contrary results because their thin-film morphology became less crystalline and denser. These results on the surface morphology in the active layers all conformed to a variation in PV performance when the DIO was incorporated. 

**Figure 5:**
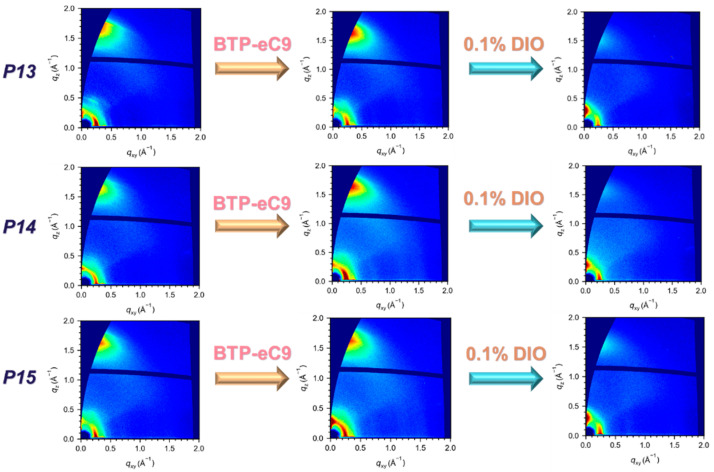
GIWAX diagrams of the thin films of the homopolymers only (**left**), the homopolymer blends/BTP-eC9 (**middle**), and homopolymer blends/BTP-eC9 with 0.1% DIO (**right**).

Furthermore, to investigate the in-plane crystallization and morphology of the thin films based on the three homopolymers, a GIWAXs analysis was applied, which revealed more detailed information about the molecular arrangement in the active layers [23,24]. As shown in Figure 5 (left), the three homopolymer-only thin films mainly formed a face-on orientation stacking arrangement, while they also had a partly edge-on-oriented molecular arrangement. After blending with BTP-eC9, as seen in Figure 5 (middle), all the homopolymers underwent significant changes. For P13 and P14, the blended thin films both showed an obvious enhancement in terms of a face-on orientation stacking arrangement. Meanwhile the P15-based devices underwent a significant increase in terms of an edge-on orientation stacking arrangement, and their face-on orientation stacking arrangement also had a slight enhancement. After incorporating 0.1% DIO (Figure 5, right), all the blended thin films became dominated by an edge-on orientation stacking arrangement. Except for P15, the face-on orientation arrangements of the others almost totally disappeared, which indicated an increase in the electron transport properties and a decrease in the hole transport properties of their OPVs. However, an excellent organic semiconductor device requires a balance between hole and electron transport properties [25].

Obviously, the P13 and P14 blended thin films had the worst balances, resulting in decreases in the *J*_SC_ values for their corresponding OPV devices after adding the additive. Meanwhile, the P15-based blended thin film showed such a better balance that its *J*_SC_ exhibited a slight increase with the incorporation of DIO in its OPV device. 

## 3. Materials and Methods

The raw materials of benzo[1,2-b:4,5-b′]dithiophene-4,8-dione, 3-chloro-2-(2-ethylhexyl)thiophene and 2-(2-ethylhexyl)thiophene were purchased from SunaTech Inc., Suzhou, China. Moreover, all other solvents and reagents were purchased from Sigma-Aldrich (Soeborg, Denmark) and used without further purification. NMR spectra were recorded at room temperature with a JEOL JNM-ECZ400S spectrometer (400 MHz for ^1^H NMR and 100 MHz for ^13^C NMR, Akishima, Japan). Elemental analysis (EA) was carried out using a Euro Vector EA3000 analyzer (Pavia, Italy). The UV–visible spectra were obtained with a JASCO V-650 UV-vis spectrometer (Tokyo, Japan). In addition, a thermo-gravimetric analysis (TGA) was carried out using the STA 449 F3 Jupiter (Netzsch, Selb, Germany)^®^. An atomic force microscope (AFM) was acquired from Smart SPM (Horiba, Kyoto, Japan). PV performance was characterized under illumination with a solar simulator under an AM of 1.5 G (100 mW·cm^−2^), and J–V curves were recorded using a Keithley 2400 source meter (Tektronix, Beaverton, OR, USA). The EQE of solar cells was analyzed using an Enlitech QE-R Quantum Efficiency Analyzer (Taiwan, China). Two-dimensional grazing-incidence wide-angle X-ray scattering (GIWAXS) patterns of films prepared at different temperatures were obtained at the 1W1A Diffuse X-ray Scattering Station, Beijing Synchrotron Radiation Facility (BSRF-1W1A). The monochromatic wavelength of the light source was 1.54 Å. The data were recorded with a Pilatus 100 K detector from DECTRIS, Baden, Switzerland. The grazing incidence angle was 0.2°.

Cyclic voltammetry (CV) of the three polymers was recorded on a CH Instruments Model 650A Electrochemical Workstation (Artisan Technology, Champaign, IL, USA). A three-electrode configuration was used, with Pt wires as both the working and counter electrodes and a freshly activated Ag wire as the Ag/Ag^+^ pseudo-reference electrode. To obtain the oxidation potentials, the reference electrode was calibrated using ferrocene/ferrocenium (Fc/Fc^+^), which has an absolute potential of 4.8 eV versus vacuum level. And a redox potential of 0.418 eV for Fc/Fc^+^ was obtained for calibration vs. an Ag/Ag^+^ electrode under the same conditions. And a tetrabutylammonium hexafluorophosphate (Bu_4_NPF_6_) solution (0.1 M of solution in anhydrous acetonitrile) was used as a supporting electrolyte, with N_2_ gas bubbled prior to each measurement. The homopolymers were deposited onto the working electrode by drop casting from CHCl_3_ solutions of 10 mg·mL^−1^. The HOMO and LUMO levels were calculated from the formulas E_HOMO_/E_LUMO_ = −(E_OX_/E_RED_ + 4.8 − |Fc/Fc^+^|) eV and E_g_ = E_LUMO_ − E_HOMO_, where E_OX_, E_RED_, E_g_^opt^, and |Fc/Fc^+^| were determined from the oxidation/reduction onsets in the CV curves, i.e., the value of 1240/λ (the onset absorption band edge of the polymer films) and the half-wave potential of ferrocene in the CV curves, respectively.

**Fabrication of OSC devices**

The OSC devices were fabricated on top of a pre-patterned ITO substrate with a conventional structure of ITO/PEDOT:PSS/active layers/PFN-Br/Ag. After cleaning the ITO glass with aqueous detergent, deionized water, acetone, and 2-propanol, respectively, UV–ozone treatment was applied for 15 min. Filtered PEDOT:PSS (CLEVIOS™ P VP AI 4083, Heraeus, Leverkusen, Germany) was spin-coated on the ITO substrate to form a 30 nm thick layer. Then, the coated substrates were annealed on thermal plates at 150 °C for 20 min. After annealing, a chlorobenzene (CB) solution of the blending active substances was spin-cast on top of the PEDOT:PSS layer to produce the active layer with a thickness of 100 nm under 2000 rpm in a glovebox, and the coated substrates with the active layer were annealed for 5 min at 100 °C. Then, PFN-Br (Poly(9,9-bis(3′-(*N*,*N*-dimethyl)-*N*-ethylammoinium-propyl-2,7-fluorene)-alt-2,7-(9,9-dioctylfluorene))dibromide) was spin-coated on the active-layer substrates under 3000 rpm. Finally, the device fabrication was completed by the thermal evaporation of 100 nm of Ag as the cathode at a high vacuum pressure (<10^−6^ mbar).

**Charge carrier mobility, dissociation, and recombination**

The hole mobilities of the active layers were measured by applying the space-charge-limited current (SCLC) method to the J–V measurements of the devices. The hole-only homopolymer devices were designed as semiconductor diodes with a structure of ITO/PEDOT:PSS/active layer/Au, and their current density was calculated from the following equation [26]:

$$J_{\mathrm{SCLC}}=\left(9/8\right)\varepsilon_{r}\varepsilon_{0}\mu \left(V^{2}/L^{3}\right)\exp\left(0.89r\sqrt{V/L}\right)$$

where J represents the current density, ε_r_ stands for the dielectric constant of the polymers, ε_0_ is the permittivity of a vacuum, μ refers to the hole mobility, L is the thickness of the blend films, V = V_appl_ − V_bi_, where V_appl_ is the applied potential, and V_bi_ is the built-in voltage, which results from the difference between the work functions of the anode and cathode.

The charge dissociation probability was characterized by the function of the photogenerated current density (J_ph_) versus the effective applied voltage (V_eff_). J_ph_ is defined as the difference between J_L_ and J_D_, where J_L_ and J_D_ are the current densities of the devices in light (100 mW·cm^−2^) and dark conditions, respectively. V_eff_ = V_0_ − V, where V_0_ is the voltage when J_ph_ = 0, and V is the applied voltage during the measurement. When the reverse voltage is greater than 2 V, recombination is suppressed by a high internal electric field. Thus, J_ph_ will reach a saturated current density (J_sat_). Consequently, P_diss_ = J_ph_/J_sat_ could be used to describe the charge dissociation probability [27,28]. And a higher P_diss_ indicates more effective charge dissociation. Furthermore, the relationship between *J*_SC_ and the light intensity (I) is *J*_SC_ ∝ I^α^, where α is the degree of biomolecular recombination. When α = 1, dissociated free charges do not recombine during the movement process and are all collected by the electrode, implying that the recombination can be ignored. If α is less than 1, bimolecular recombination will be present in the devices, and the smaller the value of α, the stronger the bimolecular recombination. Meanwhile, V_OC_ ∝ (nkT/q)·ln(I), where K represents the Boltzmann constant, T is the Kelvin temperature, and q is the elementary charge. If the slope is close to 2 kT/q, trap-assisted recombination will occur inside the devices[29,30].

## 4. Conclusions

In this work, we successfully designed and synthesized high-performance BDT-based D1-D2-type homopolymer donor materials, namely P13, P14, and P15, which achieved the highest PCE reported among OSCs based on recent analogous homopolymer donor materials. Meanwhile, we found that the solvent additive DIO induced a variation in the surfaces of the three homopolymers and the in-plane morphologies of their polymer/acceptor (BTP-eC9) blends in thin films with regard to crystallinity, phase separation, and molecular orientation. The asymmetrical structure of homopolymer P15 exhibited the best PCE of 11.5%, with an outstanding *J*_SC_ in its corresponding devices, which indicated that the asymmetric strategy had unique advantages in controlling the energy level and morphology of the polymers (as a molecular donor material for reducing unfavorable factors such as excessive phase separation and unbalanced carrier transport caused by high crystallinity and a strong aggregation effect). The P13-based asymmetrical homopolymer had a moderate PV performance owing to its large steric hindrance caused by large-sized chloroform atoms. Overall, we developed a design idea for constructing homopolymers solely based on BDT units to improve the PV performance of such D1-D2-type wide-bandgap donor materials paired with nonfullerene acceptors for cost- and resource-effective OPVs, paving the way for the large-scale production of OSC materials. 

## Figures and Tables

**Table 1:** The electrochemical and optical properties of P13, P14, and P15, obtained from solutions and thin films, respectively.

Sample	HOMO [eV] ^a^	LUMO [eV] ^a^	E_g_ [eV] ^b^	λ_edge_ [nm]	E_g_^opt^ [eV] ^c^	HOMO [eV] ^d^	LUMO [eV] ^d^
P13	−5.51	−3.63	1.88	630	1.97	−5.23	−2.18
P14	−5.53	−3.60	1.93	635	1.95	−5.27	−2.14
P15	−5.48	−3.61	1.87	640	1.94	−5.15	−2.05

^a^ Calculated from the equation E_HOMO_/E_LUMO_ = −(E_OX_/E_RED_ + 4.8 − |Fc/Fc+|) eV; ^b^ Eg = E_LUMO_ − E_HOMO_; ^c^ evaluated from the absorption band edge of the polymer films and determined by the equation E_g_^opt^ = 1240/λ(onset band edge of absorption) eV; ^d^ Calculated via DFT.

**Table 2:** The optimal PV performance of OSCs from P13, P14, and P15 as donors and BTP-eC9 as the acceptor.

Polymer/ BTP-eC9	PCE (%) ^a^	FF (%)	V_OC_ (V)	*J*_SC_ (mA·cm^−2^) ^b^
P13	9.18 (8.63)	57.10	0.88	18.33 (17.68)
P14	9.07 (8.49)	53.15	0.86	19.94 (19.33)
P15	11.53 (11.08)	65.87	0.79	22.04 (21.44)

^a^ Average PCE is calculated from 10 parallel devices. ^b^ Calculated from EQE data.

**Table 3:** The hole mobilities, charge recombination, and charge dissociation properties of P13, P14, and P15.

Items	µ_h_ (cm^2^ V^−1^ s^−1^)	α	KT/q	P_diss_ ^a^
P13:BTP-eC9	5.03 × 10^−5^	0.99	1.45	0.97
P13:BTP-eC9, 0.1% DIO	3.65 × 10^−5^	0.98	1.25	0.97
P14:BTP-eC9	1.26 × 10^−5^	0.98	1.97	0.99
P14:BTP-eC9, 0.1% DIO	7.86 × 10^−6^	0.97	1.83	0.98
P15:BTP-eC9	4.88 × 10^−5^	0.99	1.45	0.99
P15:BTP-eC9, 0.1% DIO	2.30 × 10^−4^	0.98	1.25	0.98

^a^ The charge dissociation probability (P_diss_) is described by P_diss_ = J_ph_/J_sat_. KT/q (mV), as the slope of the linear fitting curve of V_OC_ ∝ ln(I) (where K represents the Boltzmann constant, T is the Kelvin temperature, and q is the elementary charge), is a measure of the trap-assisted recombination mechanism, which favors a value of 2 KT/q for trap-assisted recombination.

## Data Availability

Data are contained within the article and Supplementary Materials.

## Supplementary Materials

The following supporting information can be downloaded at https://www.mdpi.com/article/10.3390/molecules29061332/s1: Scheme S1: Synthetic pathway of P13, P14, and P15; Figure S1: TGA curves of the three homopolymers; Figure S2: The optimization of the dimer–moiety structure of the homopolymers by DFT; Figure S3: J–V curves of P13- (A), P14- (B), and P15-based (C) OPV devices with different contents of DIO at a spin-coating speed of 2000 rpm under an illumination of AM 1.5 G, 100 mW cm^−2^. Corresponding table (bottom) of photovoltaic performance; Table S1. PV performance of BDT-based D1-D2-type homopolymer donor materials from the literature. Figure S4: Ln(JL^2^/L^3^)-(V/L)^0.5^ plots of hole-only devices based on P13:BTP-eC9, P13:BTP-eC9 (0.1% DIO), P14:BTP-eC9, P14:BTP-eC9 (0.1% DIO), P15:BTP-eC9, and P15:BTP-eC9 (0.1% DIO); Figure S5: Dependence diagram of V_OC_ (A, B) and *J*_SC_ (C, D) as a function of light intensity in OPV devices based on three homopolymers; Figure S6: P_diss_ vs. V_eff_ plots of OPV devices based on P13:BTP-eC9, P13:BTP-eC9 (0.1% DIO), P14:BTP-eC9, P14:BTP-eC9 (0.1% DIO), P15:BTP-eC9, and P15:BTP-eC9 (0.1% DIO); Figure S7: ^1^H NMR spectrum of compound **1** in CDCl_3_; Figure S8: ^13^C NMR spectrum of compound **1** in CDCl_3_; Figure S9: ^1^H NMR spectrum of compound **2** in CDCl_3_; Figure S10: ^13^C NMR spectrum of compound **2** in CDCl_3_; Figure S11: ^1^H NMR spectrum of monomer M1 in CDCl_3_; Figure S12: ^13^C NMR spectrum of monomer M1 in CDCl_3_; Figure S13: ^1^H NMR spectrum of compound **3** in CDCl_3_; Figure S14: ^13^C NMR spectrum of compound **3** in CDCl_3_; Figure S15: ^1^H NMR spectrum of monomer M3 in CDCl_3_; Figure S16: ^13^C NMR spectrum of monomer M3 in CDCl_3_; Figure S17: ^1^H NMR spectrum of compound **4** in CDCl_3_; Figure S18: ^13^C NMR spectrum of compound **4** in CDCl_3_; Figure S19: ^1^H NMR spectrum of monomer M4 in CDCl_3_; Figure S20: ^13^C NMR spectrum of monomer M4 in CDCl_3_; Figure S21: ^1^H NMR spectrum of monomer M2 in CDCl_3_; and Figure S22: ^13^C NMR spectrum of monomer M2 in CDCl_3_. References [9,31,32,33,34,35,36,37,38,39,40] are cited in the Supplementary Materials.
