# Supplementary material for: Enhanced Photovoltaic Performance of Asymmetrical Benzo Dithiophene Homopolymer Donor Materials in Nonfullerene Acceptor-Based Organic Photovoltaics
Source: Molecules. 2024 Mar 17;29(6):1332. doi: 10.3390/molecules29061332 (PMC10974560; doi:10.3390/molecules29061332)
Supplement: Supplementary file 1 [file molecules-29-01332-s001.zip › molecules-2879099-supplementary.pdf]

Electronic Supplementary Information

Enhanced Photovoltaic Performance of Asymmetrical Benzo Dithiophene Homopolymer Donor Materials in Nonfullerene Acceptor-Based Organic Photovoltaics

Wei Xu [1,2], Li Du [3], Zhengkun Du [3,*], Wei He [4], Hongxiang Li [4], Guojuan Li [5], Cheng Yang [6], Pei Cheng [4],

Zhong Cao [1,*] and Donghong Yu [2,7,*]

[1.] Hunan Provincial Key Laboratory of Materials Protection for Electric Power and Transportation, Hunan Provincial Key Laboratory of Cytochemistry, School of Chemistry and Chemical Engineering, Changsha University of Science and Technology, Changsha 410114, China

[2.] Department of Chemistry and Bioscience, Aalborg University, Fredrik Bajers Vej 7H, DK-9220 Aalborg, Denmark

[3.] College of Energy Storage Technology, Shandong University of Science and Technology, Qingdao 266590, China

[4.] State Key Laboratory of Polymer Materials Engineering, Department of College of Polymer Science and Engineering, Sichuan University, Chengdu 610065, China

[5.] National Anti-Drug Laboratory Sichuan Regional Center, Chengdu 610206, China

[6.] Key Laboratory of Green Chemistry and Technology, State Key Laboratory of Biotherapy, College of Chemistry, Sichuan University, Chengdu 610064, China

[7.] Sino-Danish Center for Education and Research, DK-8000 Aarhus, Denmark

Contents

1. Synthesis of Materials 3
2. Thermal properties and dihedral angles of the homopolymers 7
3. Fabrication and Optimization of OPV devices 8
4. Properties of charge transfer, dissociation, and recombination 10
5. Chemical structure --- NMR spectra of products 11
6. Reference 19

1. Synteses of Materials

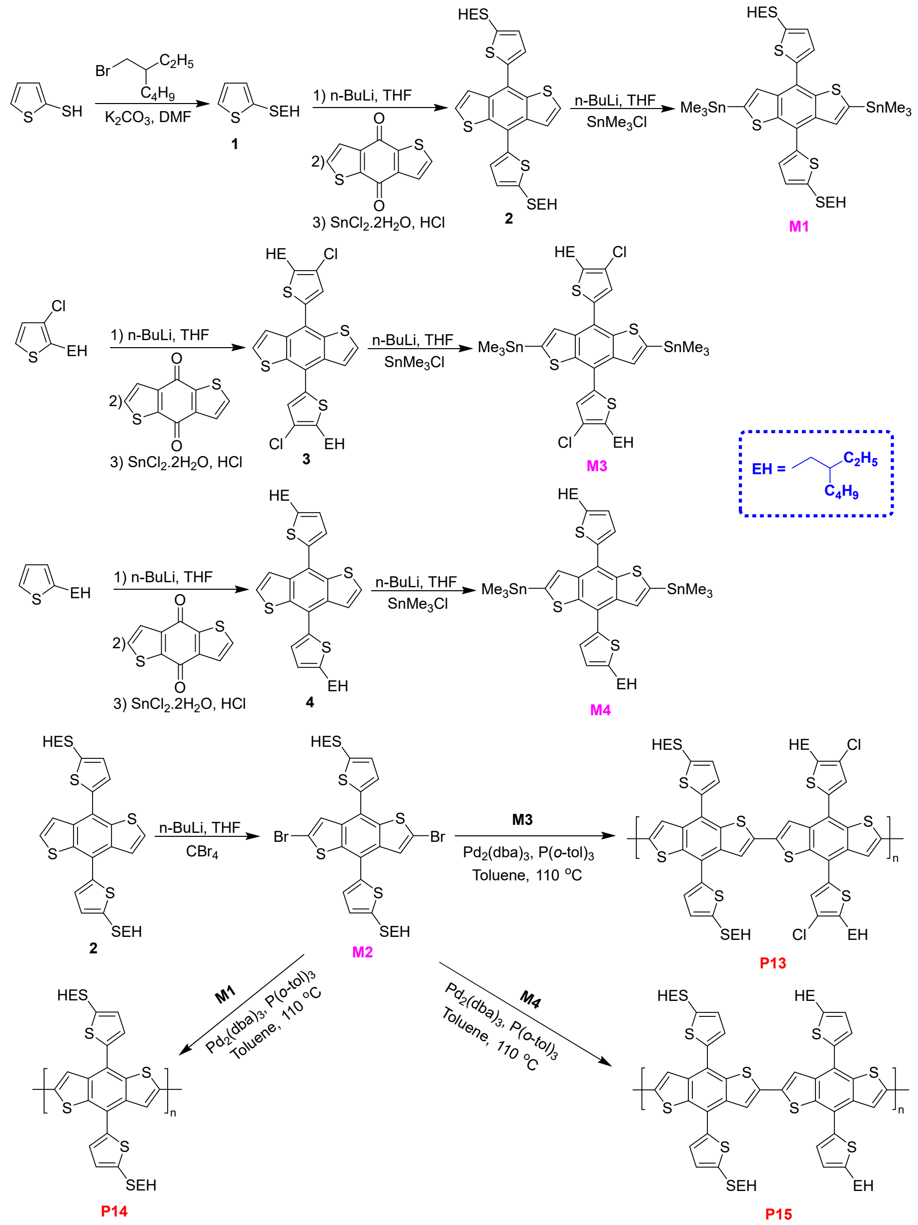

Scheme S1. *The synthetic pathway of* ***P13, P14,*** *and* ***P15****.*

2-((2-Ethylhexyl)thio)thiophene ***(1)***

Under nitrogen atmosphere, thiophene-2-thiol (2.91 g, 25 mmol), potassium carbonate (4.15 g, 30 mmol) and 1-bromo-2-ethylhexane (4.83 g, 25 mmol) were dissolved into 35 mL DMF. Then the mixture solution was stirred overnight at 100 °C. After cooling to room temperature, the reaction mixture was poured into a 200 mL water and extracted by ethyl ether three times. Subsequently, the combined organic phase was dried by anhydrous Na_2SO_4 and concentrated by rotary evaporator. The residue was purified by silica gel chromatography using petroleum ether (boiling range: 60-90 °C) as eluent to obtain compound 1 (5.25 g, yield 92%) as a pale-yellow liquid. **^{1}H NMR** (400 MHz, $CDCl_3$, ppm) δ 7.31-7.30 (dd, 1H), 7.09-7.08 (dd, 1H), 6.97-6.94 (m, 1H), 7.21-7.20 (d, 2H), 2.81-2.80 (d, 2H), 1.56-1.20 (m, 9H), 0.90-0.83 (m, 6H). **^{13}C NMR** (100 MHz, $CDCl_3$, ppm) δ 135.88, 132.65, 128.53, 127.38,4, 43.69, 38.94, 31.97, 28.68, 25.18, 22.94, 14.10, 10.67. (Figure S7 and S8)

*4,8-Bis(5-((2-ethylhexyl)thio)thiophen-2-yl)benzo[1,2-b:4,5-b']dithiophene **(2)***

Into a well-dried two-necked flask, compound 1 (2.06 g, 9.0 mmol) and 35 mL dry THF were added into under nitrogen atmosphere. The solution was cooled down to -78 °C and then 4.0 mL of n-butyllithium (9.6 mmol, 2.4 M in hexane) was added dropwise via a syringe. The mixture was stirred for 1 h at this temperature, then warmed up to 0 °C and stirred for additional 1 h. Then, the solution was cooled down to -78 °C again, Subsequently, benzo[1,2-*b*:4,5-*b*']dithiophene-4,8-dione (0.66 g, 3.0 mmol) was added into the reaction mixture in one portion quickly, and then stirred for 1.5 h at -78 °C. Then, the reaction mixture warmed up to ambient temperature slowly and stirred overnight. Then, a mixture of $SnCl_2 \cdot 2H_2O$ (5.42 g, 24 mmol) in 10%HCl (12 mL) was added and the mixture was stirred for an additional 1.5 h at 50 °C. After that, the reaction mixture was poured into water and extracted with ether three times. The combined organic phase was washed with deionized water several times and then dried over anhydrous Na_2SO_4. After the removal of solvent, the crude product was purified by column chromatography on a silica gel using petroleum ether (boiling range: 60-90 °C) as the eluent to afford compound 2 (1.64 g, yield 85%) as a yellow sticky liquid. **^{1}H NMR** (400 MHz, $CDCl_3$, ppm) δ 7.61-7.60 (d, 2H), 7.48-7.47 (d, 2H), 7.32-7.31 (d, 2H), 7.21-7.20 (d, 2H), 2.94-2.93 (d, 4H), 1.69-1.59 (m, 2H), 1.52-1.29 (m, 16H), 0.92-0.89 (t, 12H). **^{13}C NMR** (100 MHz, $CDCl_3$, ppm) δ 142.12, 138.96, 137.51, 136.48, 132.54, 128.35, 127.90, 123.60, 123.11, 43.48, 39.18, 32.09, 28.78, 25.31, 22.95, 14.15, 10.79. (Figure S9 and S10)

*(4,8-Bis(5-((2-ethylhexyl)thio)thiophen-2-yl)benzo[1,2-b:4,5-b']dithiophene-2,6-diyl)bis(trimethylstannane) **(M1)***

Compound 2 (1.29 g, 2.0 mmol) was dissolved into 45 mL dry THF at -78 °C under nitrogen protection, 2.4 mL of n-butyllithium (5.76 mmol, 2.4 M in hexane) was added dropwise. The reaction mixture was then stirred at -78 °C for 2.5 h. Subsequently, trimethyltin chloride (1.0 M in hexane, 6.5 mL) was added dropwise slowly

at -78 ^{o}C and the mixture was stirred for 6 h at room temperature. Then, the mixture was extracted with diethyl ether three times, the combined organic phase was washed with deionized water several times, and dried over anhydrous Na_2SO_4. After the removal of solvent, the crude product was purified by recrystallization using ethanol to obtain the target monomer M1 (1.34 g, yield 69%) as a light-yellow solid. **^{1}H NMR** (400 MHz, $CDCl_3$, ppm) δ 7.67-7.62 (t, 2H), 7.34 (d, 2H), 7.23-7.22 (d, 2H), 2.95-2.94 (d, 4H), 1.69-1.63 (m, 2H), 1.54-1.39 (m, 8H), 1.35-1.27 (m, 8H), 0.93-0.89 (m, 12H), 0.45-0.36 (t, 18H). **^{13}C NMR** (100 MHz, $CDCl_3$, ppm) δ 143.28, 143.04, 143.01, 137.31, 137.01, 132.60, 130.74, 128.22, 121.93, 43.54, 39.24, 32.12, 28.76, 25.35, 22.95, 14.14, 10.84, -8.31. (Figure S11 and S12)

4,8-Bis(4-chloro-5-(2-ethylhexyl)thiophen-2-yl)benzo[1,2-b:4,5-b']dithiophene ***(3)***

The target compound 3 was synthesized similarly as described above for compound 2. Compound 3 (1.52 g, yield 78%) was obtained as a light-yellow solid. **^{1}H NMR** (400 MHz, $CDCl_3$, ppm) δ 7.62-7.60 (d, 2H), 7.50-7.48 (d, 2H), 7.23 (s, 2H), 2.85-2.83 (d, 4H), 1.78-1.69 (m, 2H), 1.50-1.29 (m, 16H), 0.97-0.90 (m, 12H). **^{13}C NMR** (100 MHz, $CDCl_3$, ppm) δ 138.97, 138.31, 136.46, 135.86, 128.05, 127.97, 123.24, 123.02, 122.69, 40.89, 32.46, 32.02, 28.78, 25.76, 23.01, 14.15, 10.86. (Figure S13 and S14)

(4,8-Bis(4-chloro-5-(2-ethylhexyl)thiophen-2-yl)benzo[1,2-b:4,5-b']dithiophene-2,6-diyl)bis(trimethylstannane) ***(M3)***

The target monomer M3 was synthesized similarly as described above for monomer M1. Monomer M3 (1.65 g, yield 85%) was obtained as a light-yellow solid. **^{1}H NMR** (400 MHz, $CDCl_3$, ppm) δ 7.67-7.60 (m, 2H), 7.25 (s, 2H), 2.85 (dd, 4H), 1.80-1.71 (m, 2H), 1.50-1.31 (m, 16H), 0.99-0.90 (m, 12H), 0.49-0.35 (m, 18H). **^{13}C NMR** (100 MHz, $CDCl_3$, ppm) δ 143.29, 143.20, 137.94, 137.25, 136.62, 130.58, 127.72, 122.51, 121.50, 40.82, 32.47, 32.07, 28.81, 25.85, 23.04, 14.18, 10.91, -8.29. (Figure S15 and S16)

4,8-Bis(5-(2-ethylhexyl)thiophen-2-yl)benzo[1,2-b:4,5-b']dithiophene ***(4)***

The target compound 4 was synthesized similarly as described above for compound 2. Compound 4 (1.40 g, yield 81%) was obtained as a pale-yellow sticky liquid. **^{1}H NMR** (400 MHz, $CDCl_3$, ppm) δ 7.65-7.64 (d, 2H), 7.46-7.44 (d, 2H), 7.30-7.29 (d, 2H), 6.89 (d, 2H), 2.87-2.85 (d, 4H), 1.73-1.64 (m, 2H), 1.49-1.29 (m, 16H), 0.97-0.90 (m, 12H). **^{13}C NMR** (100 MHz, $CDCl_3$, ppm) δ 145.74, 139.05, 137.26, 136.55, 127.72, 127.45, 125.37, 124.12, 123.43, 41.52, 34.31, 32.55, 28.96, 25.79, 23.03, 14.15, 10.94. (Figure S17 and S18)

(4,8-Bis(5-(2-ethylhexyl)thiophen-2-yl)benzo[1,2-b:4,5-b']dithiophene-2,6-diyl)bis(trimethylstannane) ***(M4)***

The target monomer M4 was synthesized similarly as described above for monomer M1. Monomer M4 (1.18 g, yield 65%) was obtained as a yellow solid. **^{1}H NMR** (600 MHz, $CDCl_3$, ppm) δ 7.71-7.66 (t, 2H), 7.32-7.31 (d,

2H), 6.91-6.90 (d, 2H), 2.91-2.84 (m, 4H), 1.73-1.67 (m, 2H), 1.50-1.32 (m, 16H), 0.97-0.91 (m, 12H), 0.45-0.35 (t, 18H). **^{13}C NMR** (100 MHz, $CDCl_3$, ppm) δ 145.38, 143.27, 142.23, 138.00, 137.31, 131.17, 127.52, 125.28, 122.40, 41.49, 34.32, 32.52, 28.96, 25.82, 23.05, 14.19, 10.98, -8.36. (Figure S19 and S20)

*2,6-Dibromo-4,8-bis(5-((2-ethylhexyl)thio)thiophen-2-yl)benzo[1,2-b:4,5-b']dithiophene **(M2)***

Under nitrogen atmosphere, compound 2 (1.29 g, 2.0 mmol) was dissolved into 40 mL dry THF. Then, the mixture solution was cooled down to -78 ^{o}C and 2.4 mL of n-butyllithium (5.76 mmol, 2.4 M in hexane) was added dropwise. After the addition, the mixture was then stirred at -78 ^{o}C for 2.5 h. Subsequently, carbon tetrabromide (2.12 g, 6.4 mmol) dissolved into 10 mL dry THF was added dropwise slowly at -78 ^{o}C and the mixture was stirred overnight at room temperature. Then, the mixture was extracted with diethyl ether three times, the combined organic phase was washed with deionized water several times, and dried over anhydrous Na_2SO_4. After the removal of solvent, the crude product was purified by the silica gel column chromatography with the petroleum ether (boiling range: 60-90 ^{o}C) to obtain the target monomer M2 (1.04 g, yield 65%) as a light-yellow solid. **^{1}H NMR** (400 MHz, $CDCl_3$, ppm) δ 7.55 (s, 2H), 7.24-7.23 (d, 2H), 7.19-7.18 (d, 2H), 2.94-2.92 (dd, 4H), 1.68-1.58 (m, 2H), 1.54-1.39 (m, 8H), 1.35-1.25 (m, 8H), 0.93-0.89 (m, 12H). **^{13}C NMR** (100 MHz, $CDCl_3$, ppm) δ 140.68, 140.15, 138.46, 135.92, 132.47, 128.47, 125.78, 121.93, 117.23, 43.42, 39.18, 32.09, 28.78, 25.30, 22.95, 14.16, 10.79. (Figure S21 and S22)

*Synthesis of PBDTTS-BDTT-Cl **(P13)**, PBDTTS **(P14)** and PBDTTS-BDTT **(P15)***

Under argon atmosphere, M2 (0.1201 g, 0.15 mmol), M3 (0.1460 mg, 0.15 mmol), $Pd_2(dba)_3$ (2.7 mg, 0.0029 mmol) and P(*o*-tol)$_3$ (5.4 mg, 0.0177 mmol) were dissolved in dry toluene (6 mL) and DMF (1 mL), and then the mixture was refluxed at 110 °C for 8 h. After cooling to room temperature, the reaction was filtered in methanol. Then the precipitate was extracted by using acetone, hexane and DCM with Soxhlet extraction. The crude product was dissolved in 100 mL hot chlorobenzene, and purified via silica gel flash column with a column length of about 5 cm after cooling to r.t, using chlorobenzene as the eluent. Then the concentrated chlorobenzene phase was filtrated in methanol, and vacuum-dried at 45 °C overnight to obtain the target homopolymer P13. Besides, asymmetric homopolymers, P14 and P15, were synthesized via the same ways.

2. Thermal properties and dihedral angles of the homopolymers

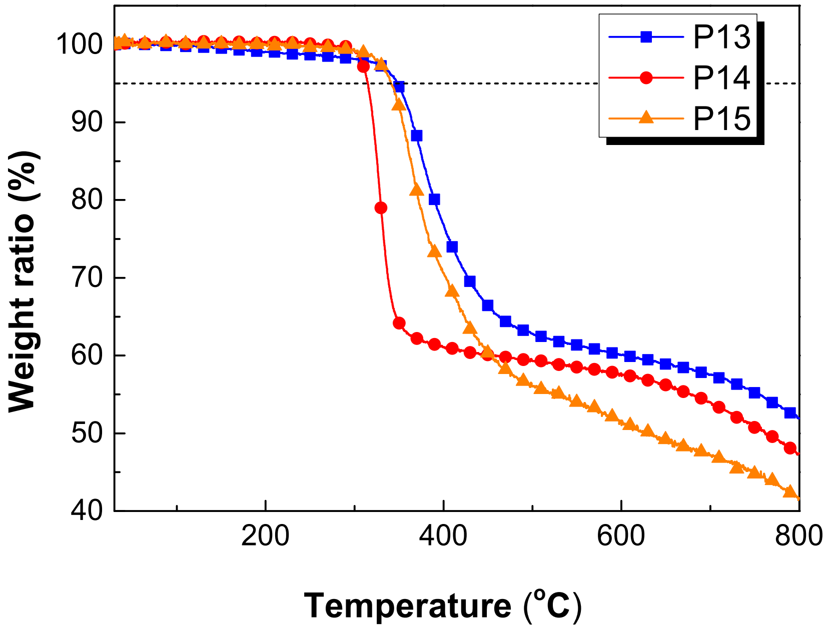

Figure S1. TGA curves of three homopolymers.

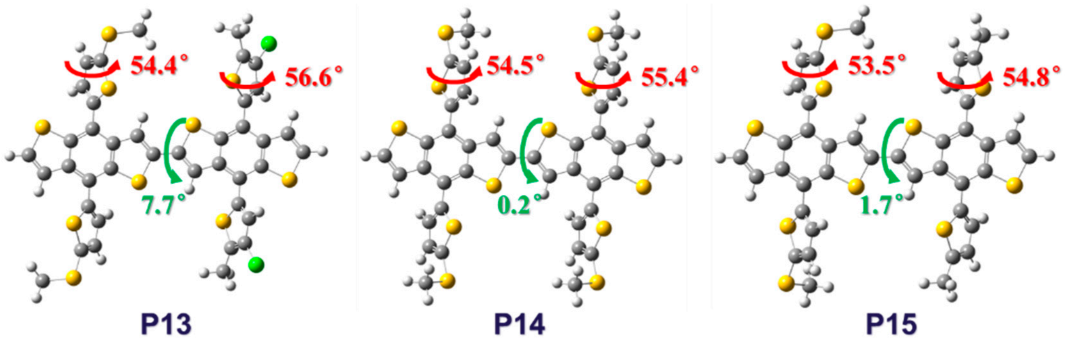

Figure S2. The optimized dimer-moieties structure of the homopolymers by DFT.

3. Fabrication and Optimization of OPV devices

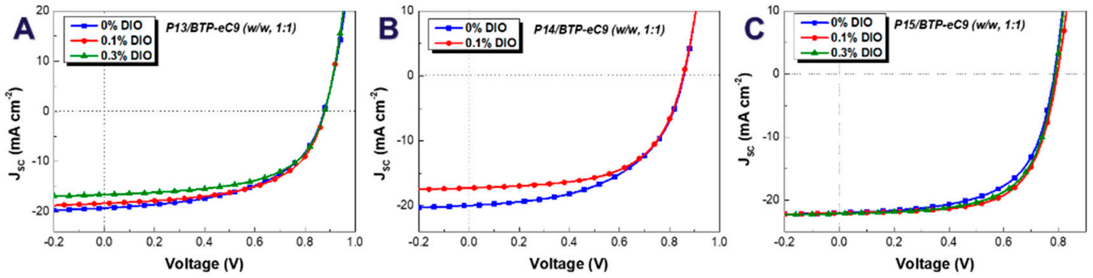

Polymer/ BTP-eC9	D:A	DIO (vol%)	PCE (%)	FF (%)	V_{oc} (V)	J_{sc} (mA cm^{-2})
P13	1:1	0	8.92	52.90	0.88	19.25
		0.1	9.18	57.10	0.88	18.33
		0.3	8.46	58.24	0.88	16.58
P14	1:1	0	9.07	53.15	0.86	19.94
		0.1	8.76	59.54	0.85	17.26
P15	1:1	0	10.61	61.69	0.78	21.97
		0.1	11.53	65.87	0.79	22.04
		0.3	11.20	64.46	0.79	22.10

Figure S3. J-V curves of P13- (A), P14- (B) and P15-based (C) OPV devices with different content of DIO at spin-coating speed of 2000 rpm under an illumination of AM 1.5 G, 100 mW cm^{-2}. Corresponding table (bottom) of Photovoltaic performance.

Table S1. PV performance of BDT based D1-D2 type homopolymer donor materials from literatures.

Polymer/acceptor	E_g^{opt} (eV)	V_{oc} (V)	J_{sc} (mA·cm^{-2})	FF (%)	PCE (%)	Ref.
PBDTT/$PC_{71}BM$	2.13	0.93	11.95	55.00	6.12	[1]
PBDTPBI*	2.10	0.69	7.54	53.00	2.73	[2]
S-PBDTPBI*	2.08	0.78	8.05	57.00	3.60	[2]
SF-PBDTPBI*	2.08	0.92	7.60	60.00	4.18	[2]
PBDTT/PBI	2.13	0.93	2.17	46.00	0.94	[2]
TT-BDT6T/$PC_{61}BM$	1.78	0.97	9.40	61.79	5.64	[3]
TS-BDT6T/$PC_{61}BM$	1.75	0.96	8.47	60.50	4.89	[3]

TB-BDT6T/$PC_{61}BM$	1.76	0.85	6.87	58.72	3.41	[3]
PffBDT4T-2OD/$PC_{71}BM$	1.44	0.79	17.75	73.00	10.23	[4]
PBDT[2H]T/ITIC	2.10	0.79	7.60	41.0	2.50	[5]
PBDT[2F]T/ITIC	2.10	0.94	11.6	53.0	5.80	[5]
PBDT(T)[2F]T/ITIC	2.00	0.94	16.9	62.0	9.80	[5]
HD-PBDT2 FT/ITIC	2.00	0.92	14.4	65.00	8.7	[6]
OD-PBDT2 FT/ITIC	2.00	0.92	13.1	69.00	8.3	[6]
DT-PBDT2 FT/ITIC	2.00	0.95	13.1	57.20	7.0	[6]
POTTF2T/ITIC	1.86	0.60	6.78	45.60	1.85	[7]
PSTTF2T/ITIC	1.89	0.93	9.80	57.40	5.22	[7]
PBDT-2TC/ITIC	1.96	0.93	15.3	65.70	9.35	[8]
PBDT-S-2TC/ITIC	1.94	0.96	16.4	64.30	10.12	[8]
PBDT-TT/IT-M	1.88	0.895	17.90	71.00	11.38	[9]
PBDT-BDTC/ITIC-Th	1.90	0.92	13.91	65.21	8.32	[10]
PBDTS-BDTC/ITIC-Th	1.90	0.97	13.94	57.73	7.49	[10]
PBDTT/IDIC	2.13	0.88	13.70	43.90	5.30	[11]
PBBF/IDIC	2.09	0.95	15.30	58.80	8.50	[11]
P13	1.97	0.88	18.33	57.10	9.18	This work
P14	1.95	0.86	19.94	53.15	9.07	This work
P15	1.94	0.79	22.04	65.87	11.53	This work

*Single component materials based OSCs without separated electron-acceptor materials included.

4. Properties of charge transfer, dissociation, and recombination

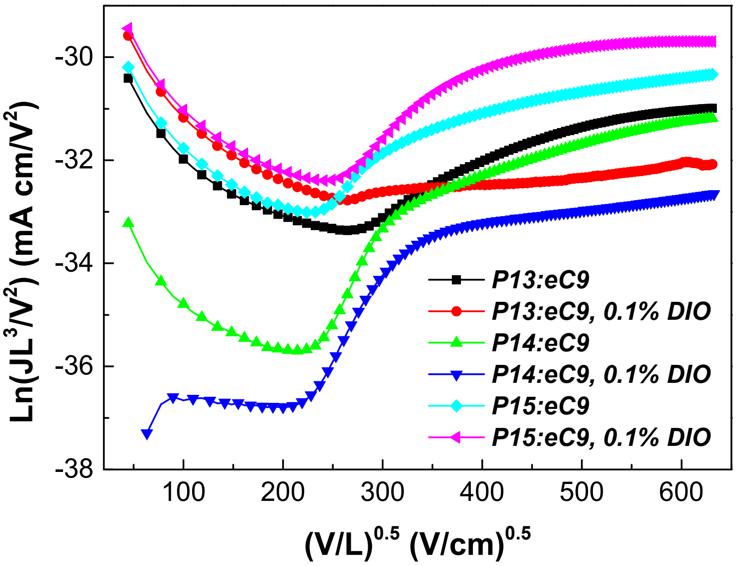

Figure S4. Ln(JL^2/L^3)-(V/L)$^{0.5}$ plots of hole-only devices based on P13:BTP-eC9, P13:BTP-eC9 (0.1% DIO), P14:BTP-eC9, P14:BTP-eC9 (0.1% DIO), P15:BTP-eC9, P15:BTP-eC9 (0.1% DIO).

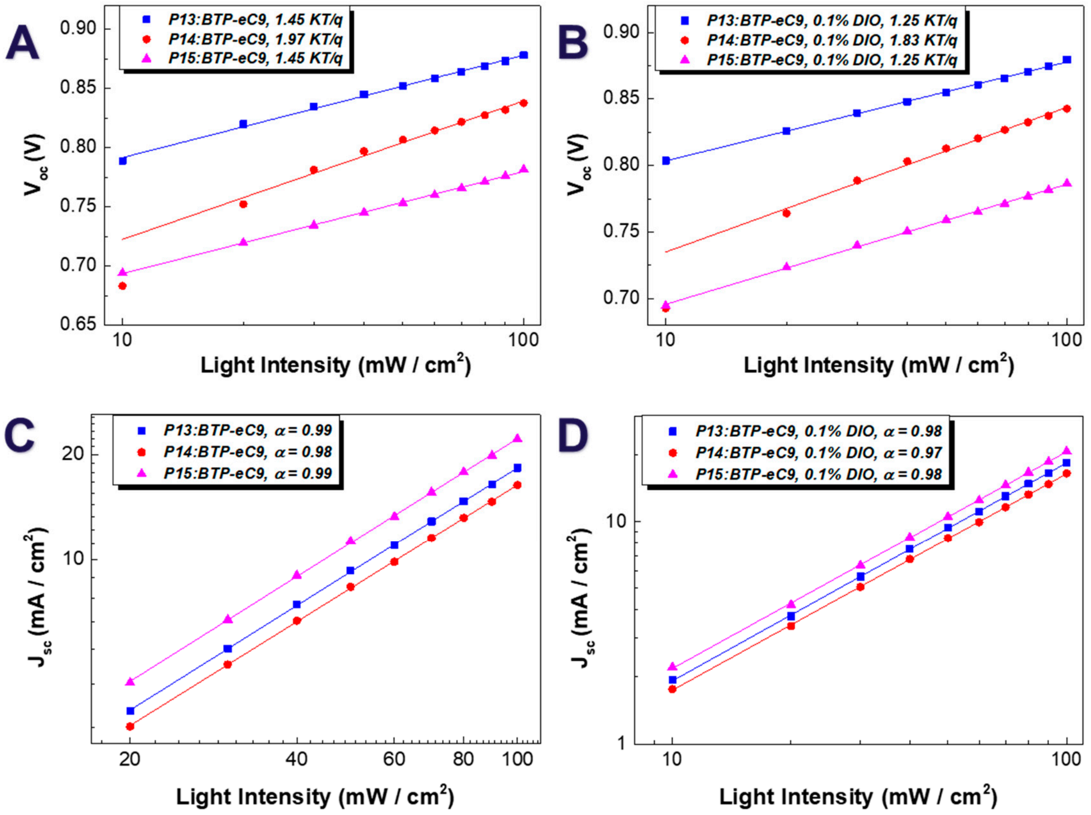

Figure S5. Dependence diagram of V_{OC} (A, B) and J_{SC} (C, D) as a function of light intensity in OPV devices based on three homopolymers.

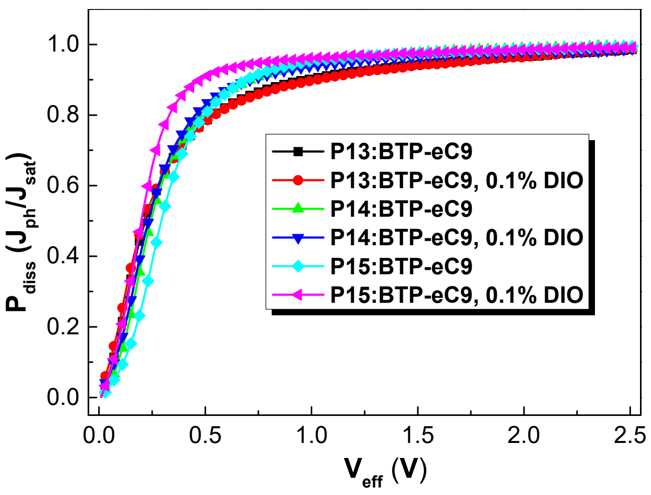

Figure S6. P_{diss} vs V_{eff} Plots of OPV devices based on P13:BTP-eC9, P13:BTP-eC9 (0.1% DIO), P14:BTP-eC9, P14:BTP-eC9 (0.1% DIO), P15:BTP-eC9, P15:BTP-eC9 (0.1% DIO).

5. Chemical structure --- NMR spectra of products

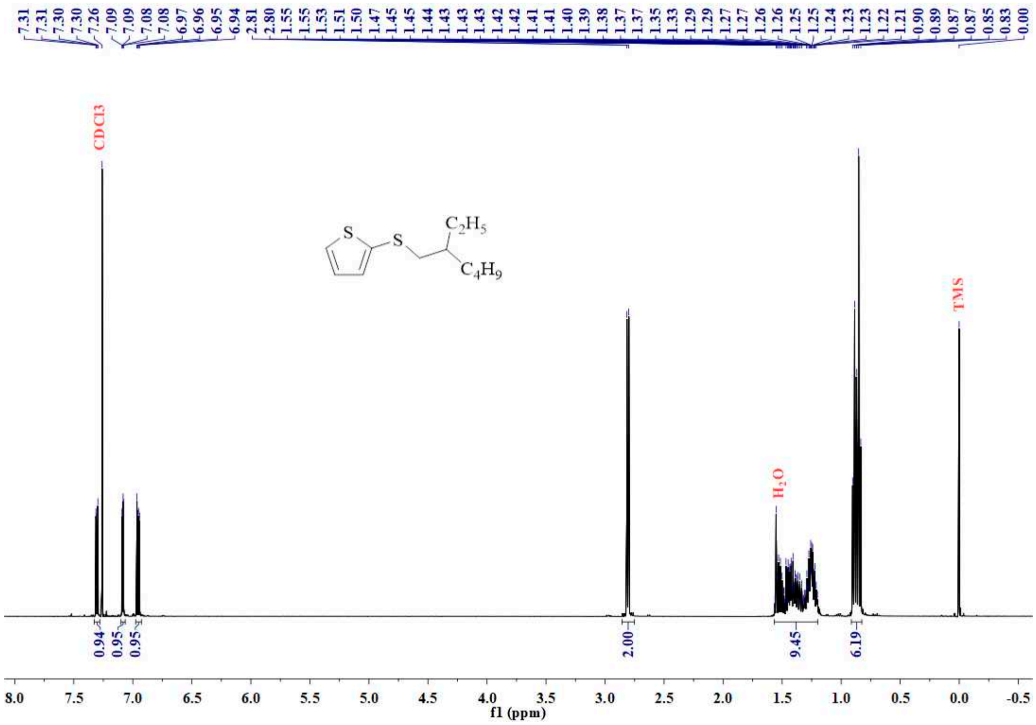

Figure S7. ^{1}H NMR spectrum of compound 1 in $CDCl_3$.

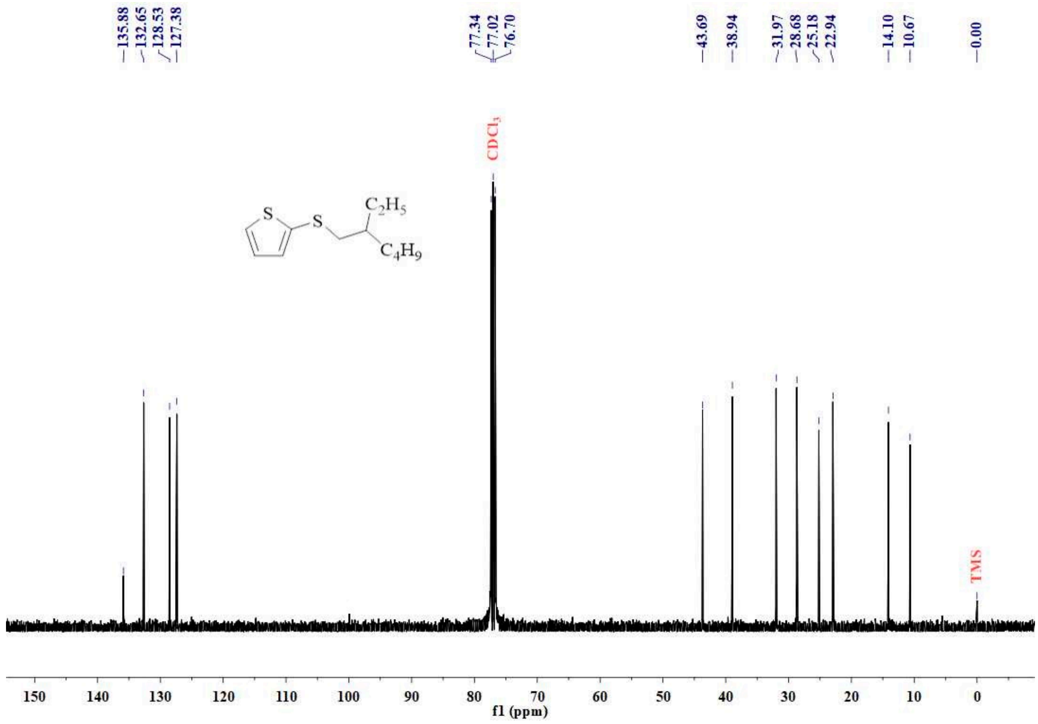

Figure S8. ^{13}C NMR spectrum of compound 1 in $CDCl_3$.

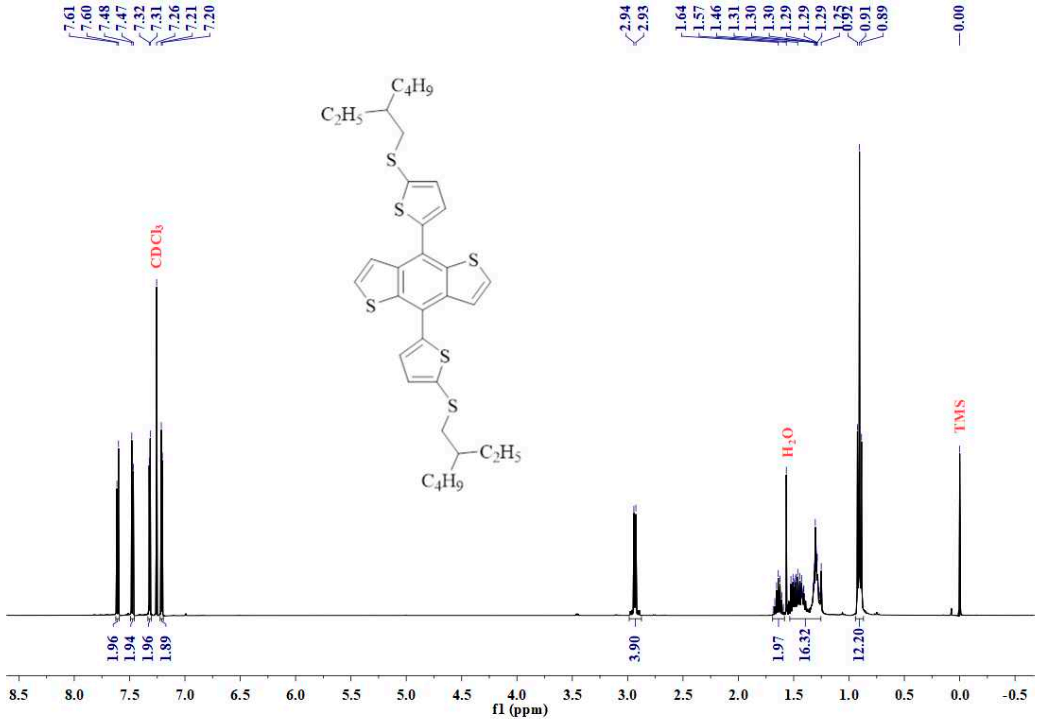

Figure S9. ^{1}H NMR spectrum of compound 2 in $CDCl_3$.

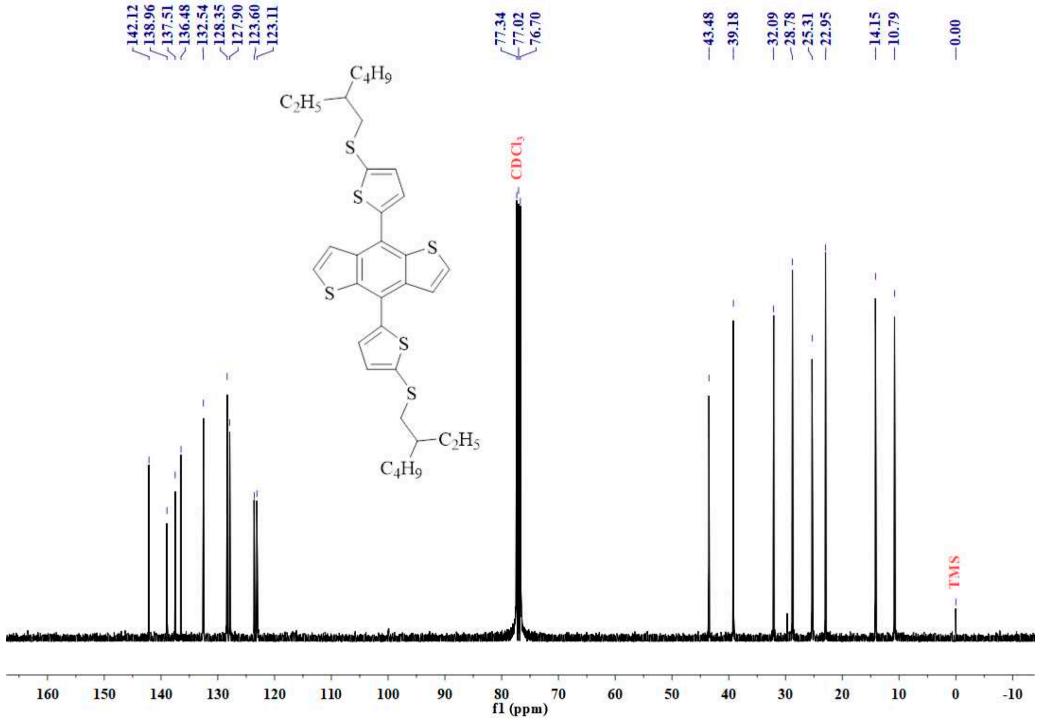

Figure S10. ^{13}C NMR spectrum of compound 2 in $CDCl_3$.

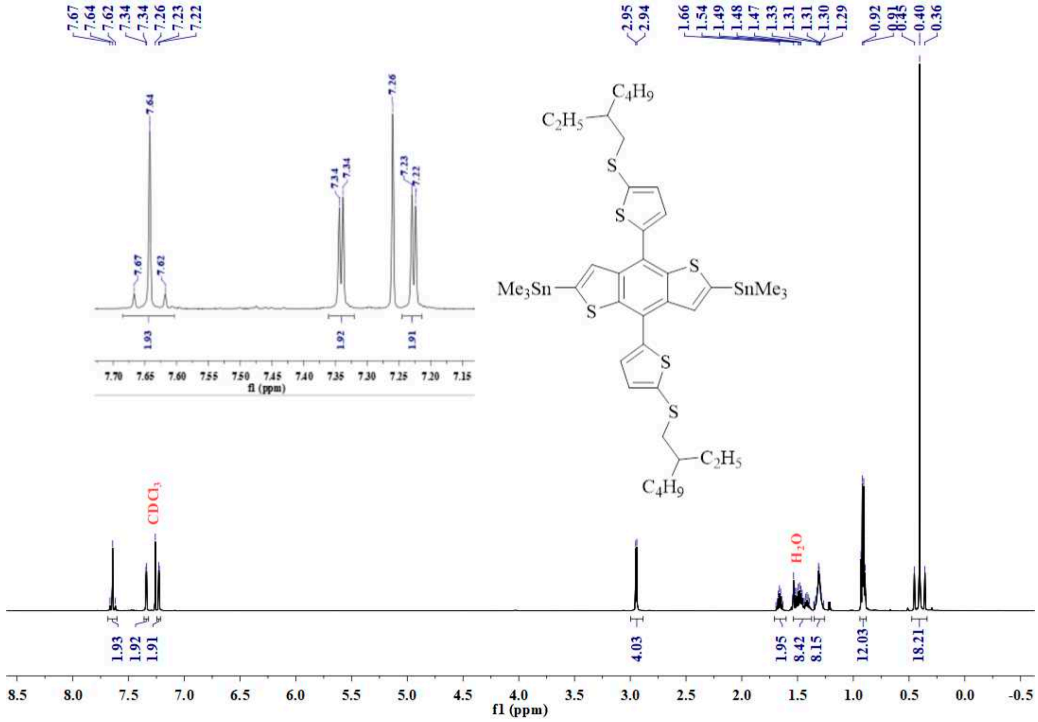

Figure S11. ^{1}H NMR spectrum of monomer M1 in $CDCl_3$.

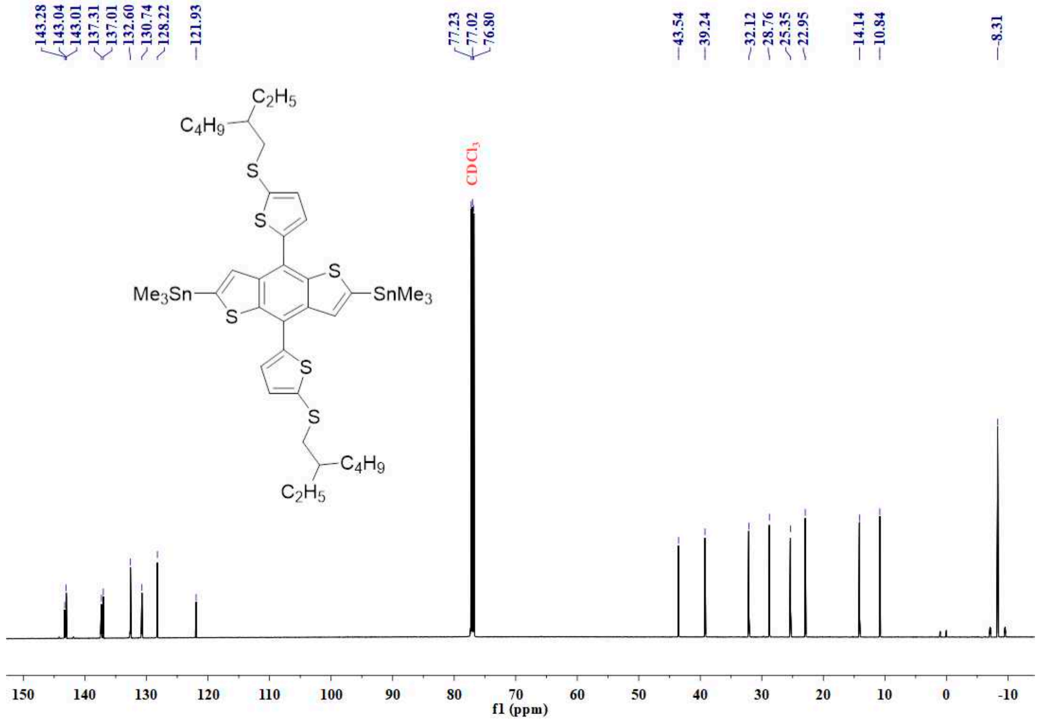

Figure S12. ^{13}C NMR spectrum of monomer M1 in $CDCl_3$.

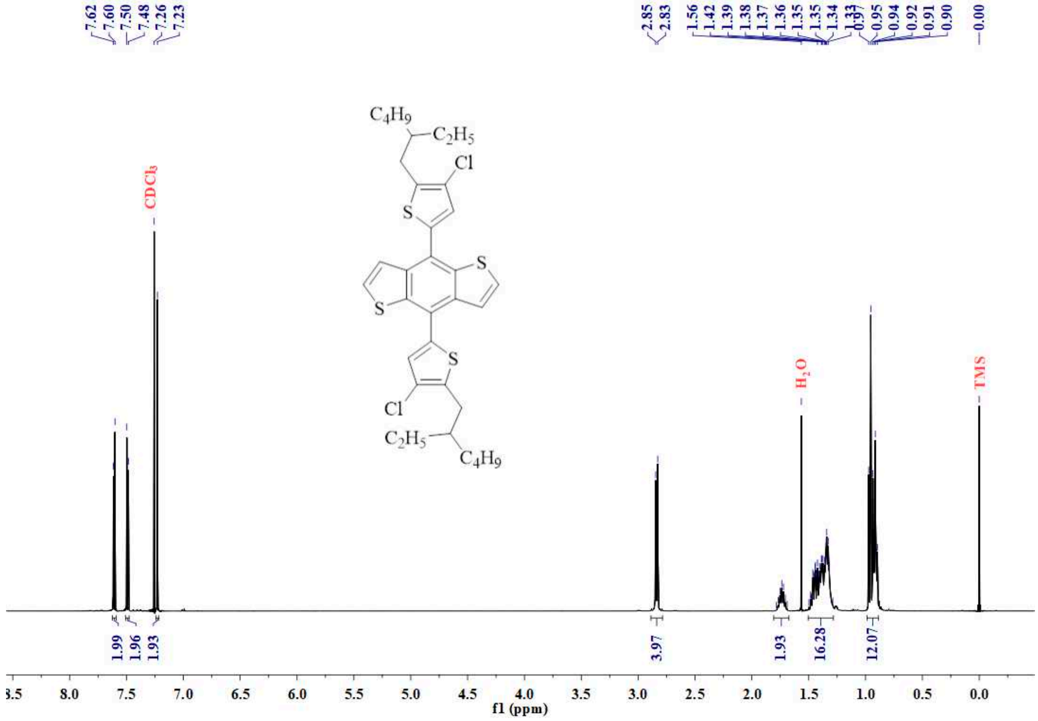

Figure S13. ^{1}H NMR spectrum of compound 3 in $CDCl_3$.

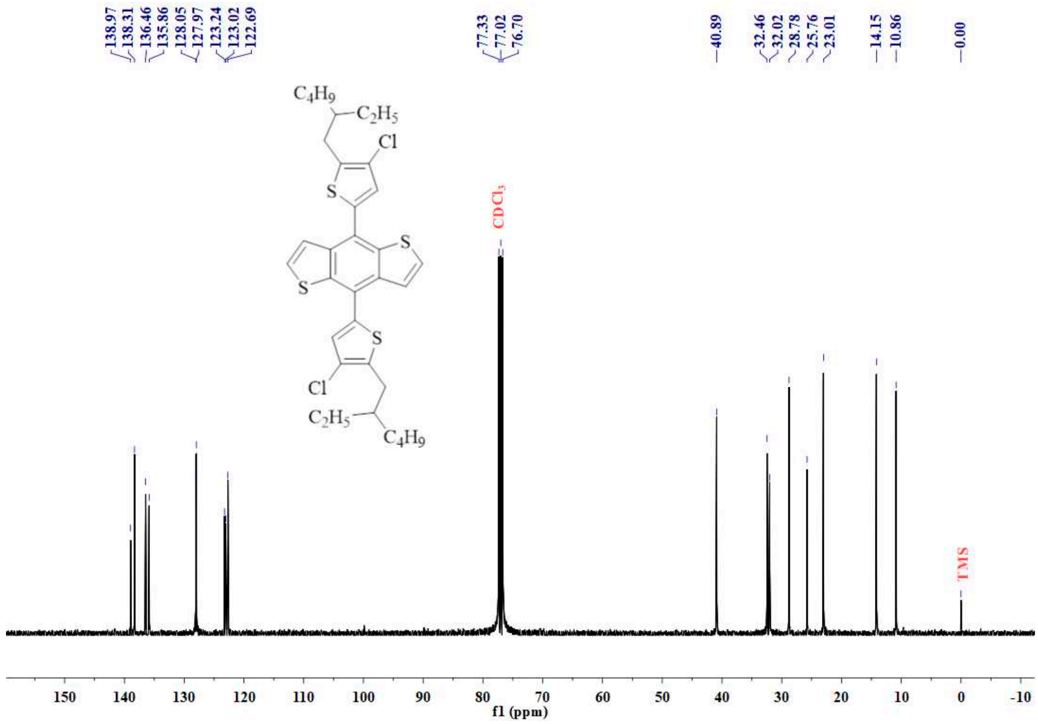

Figure S14. ^{13}C NMR spectrum of compound 3 in $CDCl_3$.

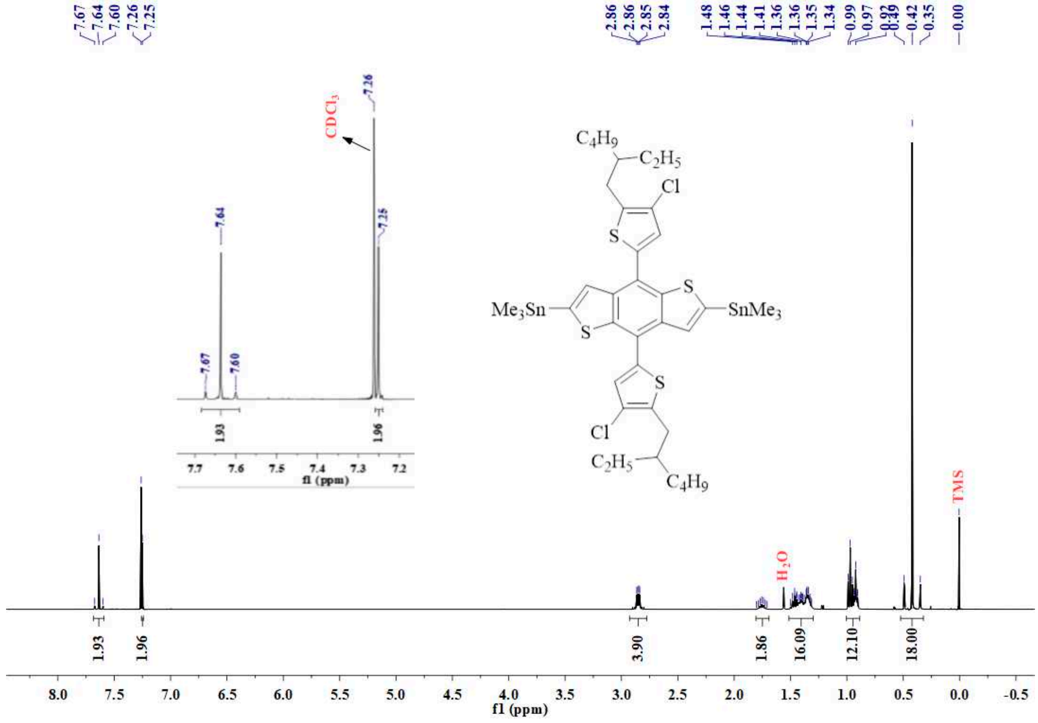

Figure S15. ^{1}H NMR spectrum of monomer M3 in $CDCl_3$.

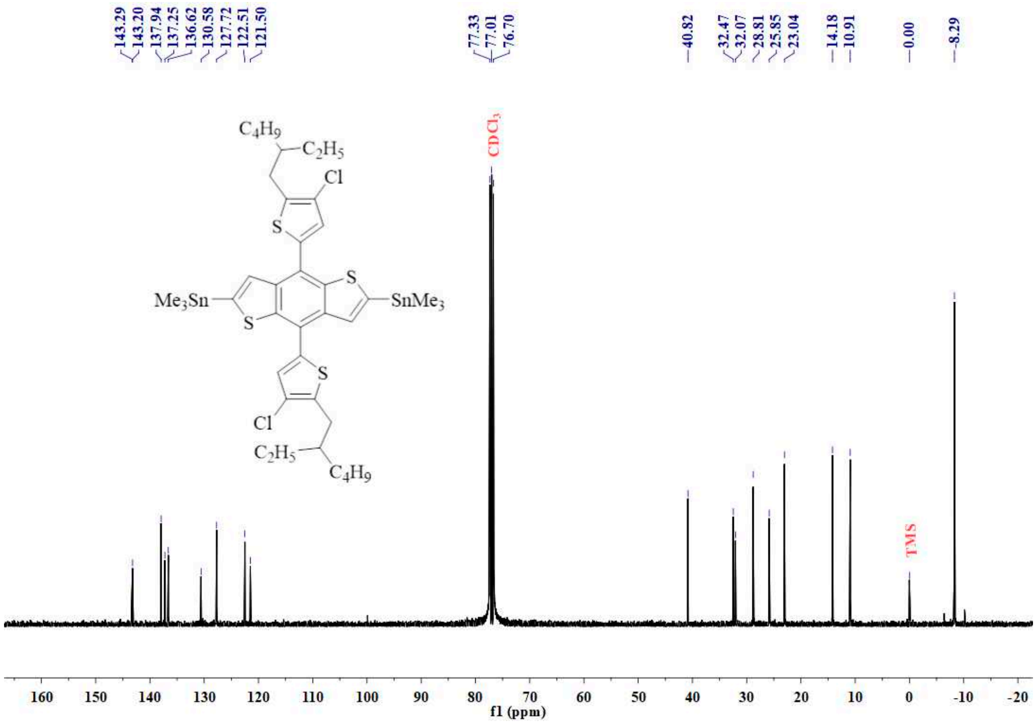

Figure S16. ^{13}C NMR spectrum of monomer M3 in $CDCl_3$.

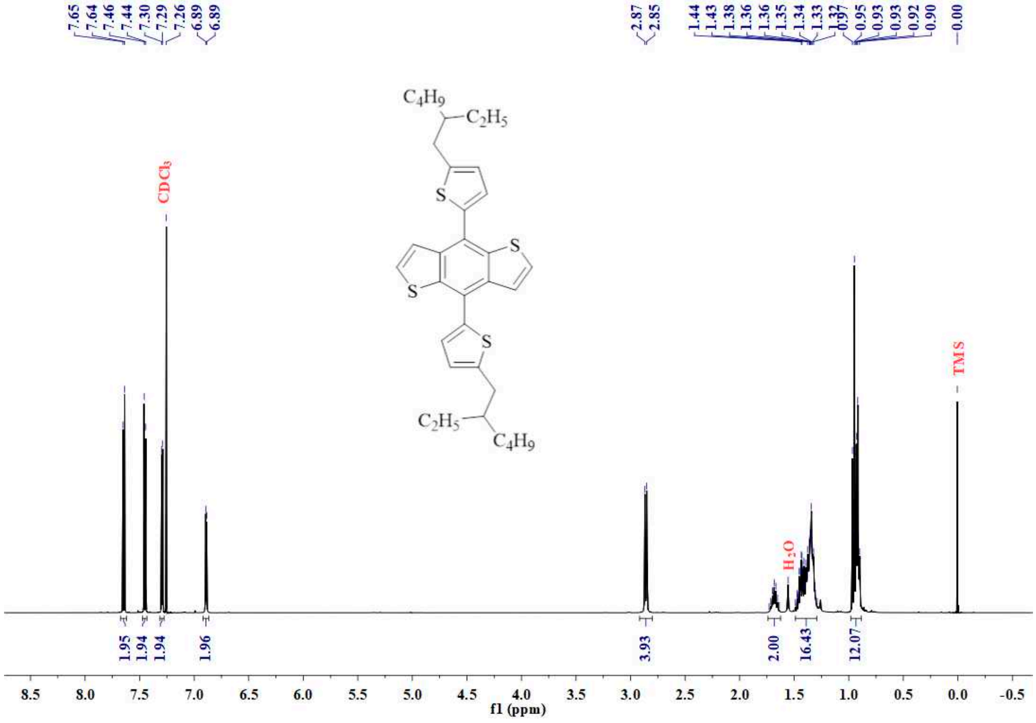

Figure S17. ^{1}H NMR spectrum of compound 4 in $CDCl_3$.

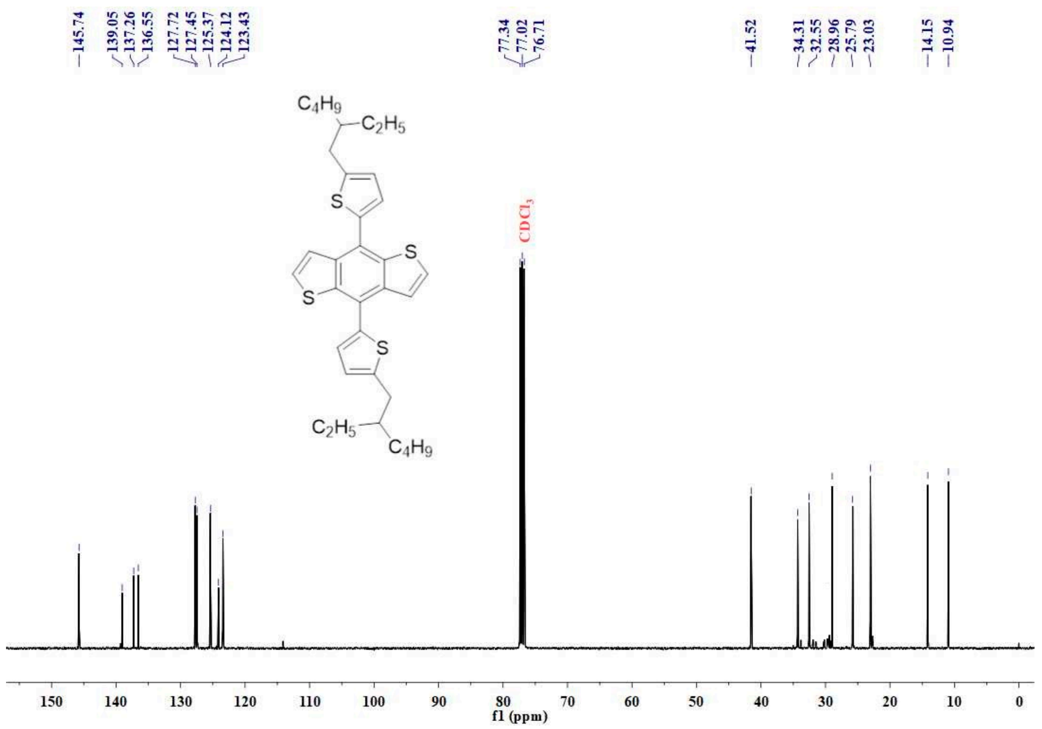

Figure S18. ^{13}C NMR spectrum of compound 4 in $CDCl_3$.

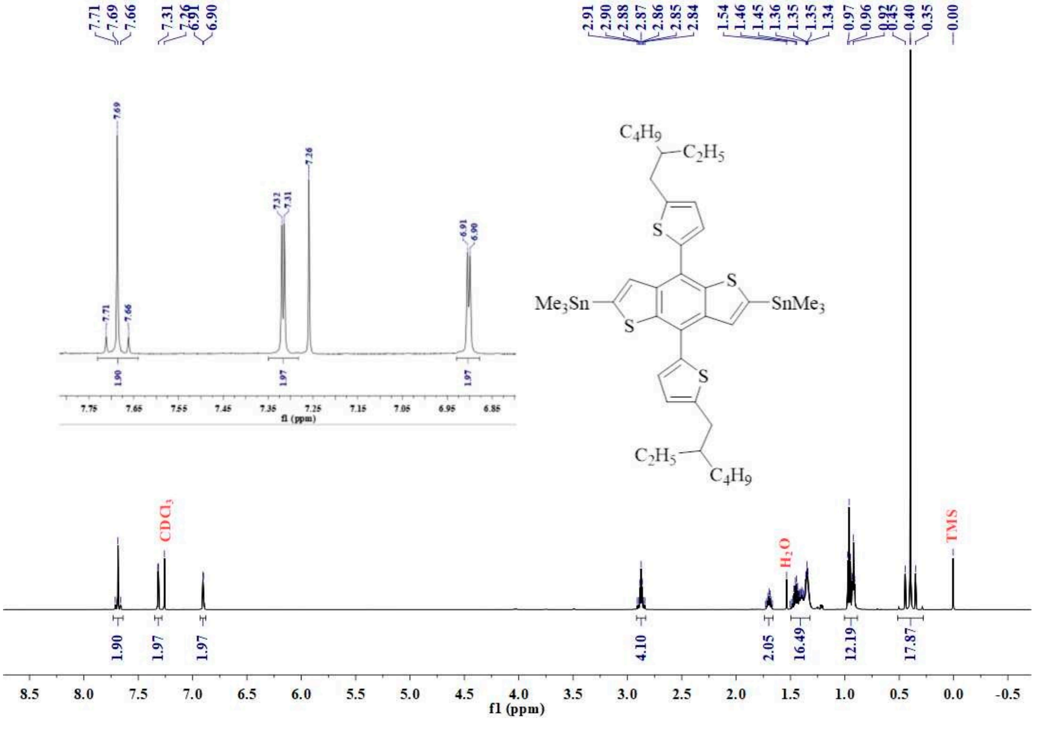

Figure S19. ^{1}H NMR spectrum of monomer M4 in $CDCl_3$.

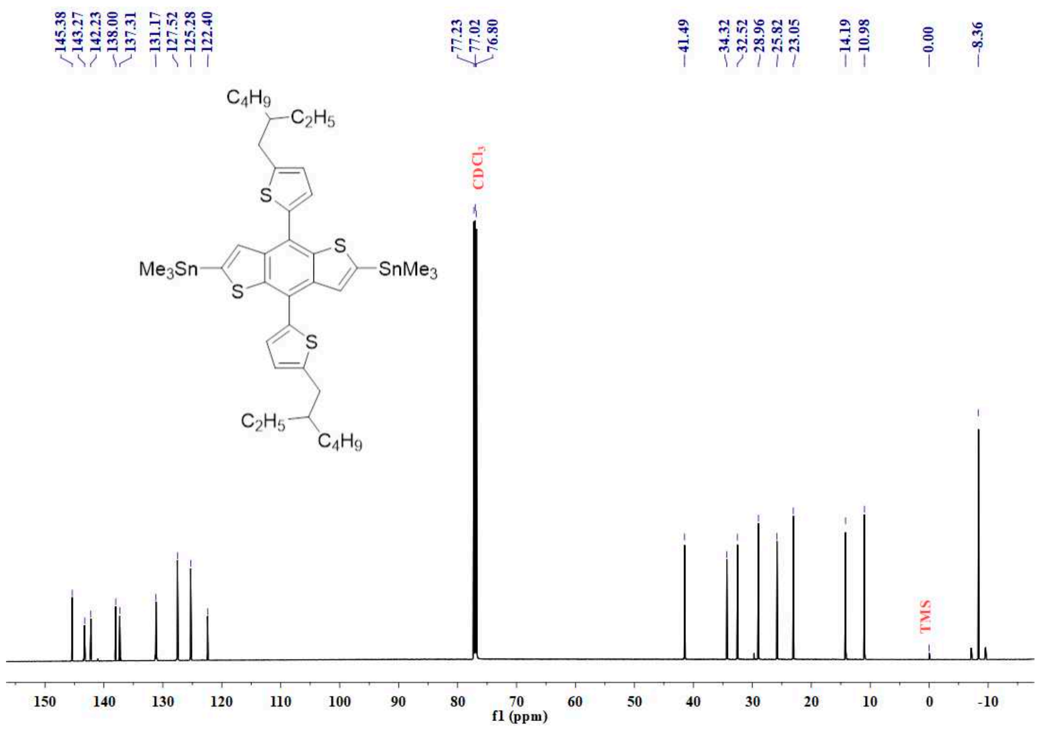

Figure S20. ^{13}C NMR spectrum of monomer M4 in $CDCl_3$.

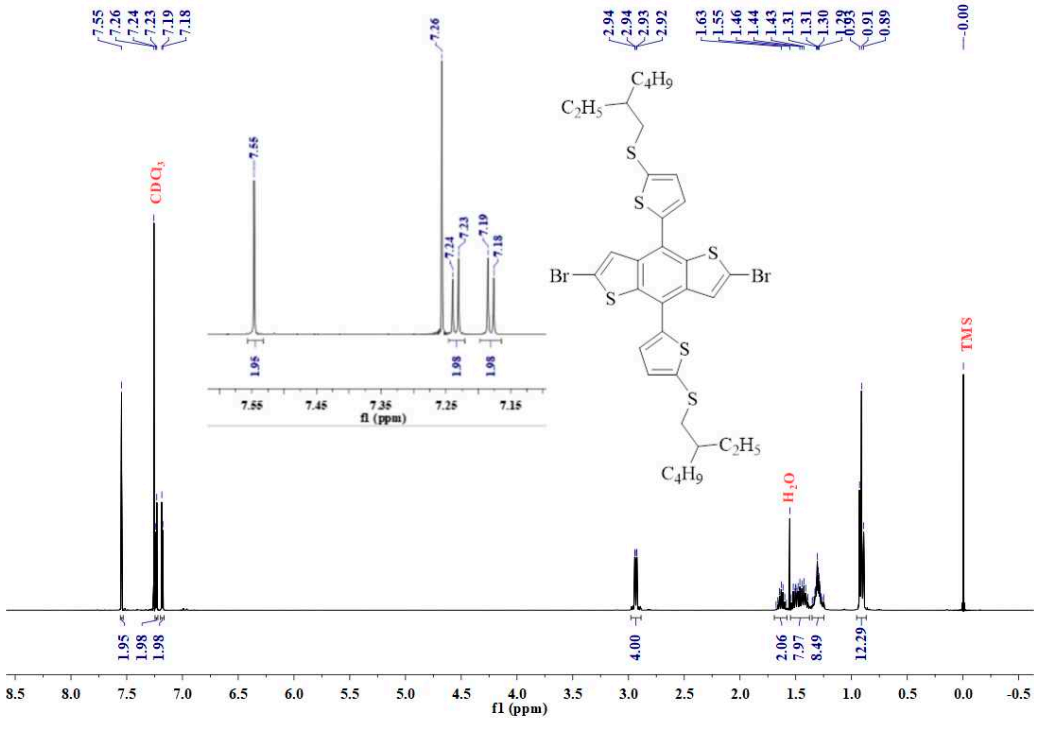

Figure S21. ^{1}H NMR spectrum of monomer M2 in $CDCl_3$.

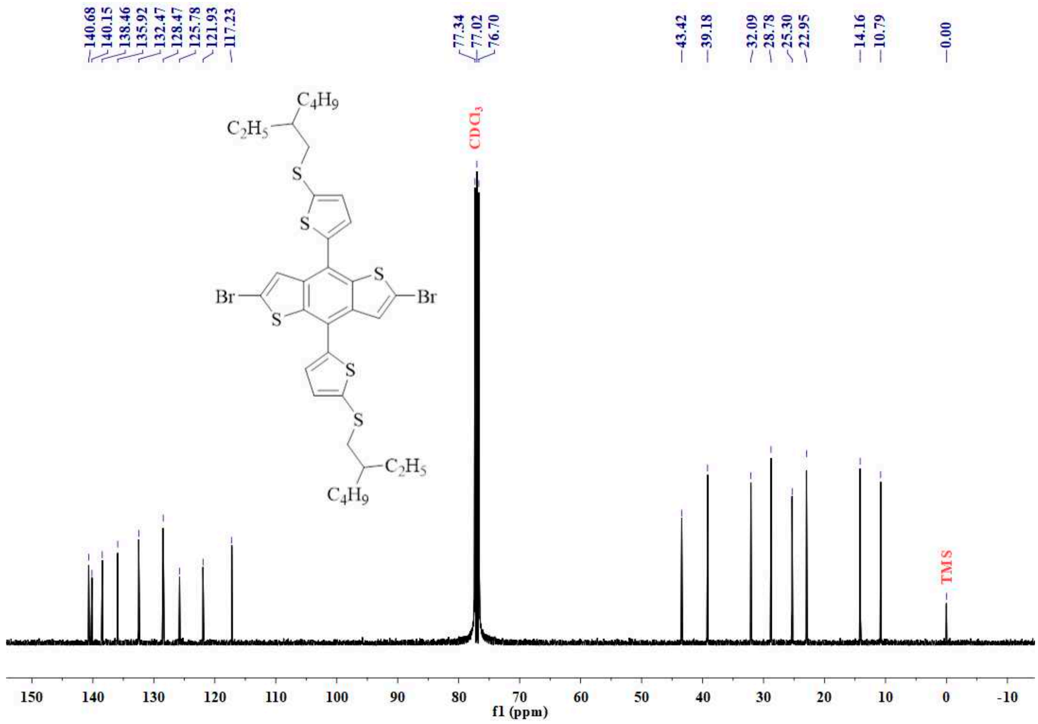

Figure S22. ^{13}C NMR spectrum of monomer M2 in $CDCl_3$.

6. Reference

1. Kang, T.E.; Kim, T.; Wang, C.; Yoo, S.; Kim, B.J. Poly(Benzodithiophene) Homopolymer for High-Performance Polymer Solar Cells with Open-Circuit Voltage of Near 1 V: A Superior Candidate To Substitute for Poly(3-Hexylthiophene) as Wide Bandgap Polymer. *Chem Mater* **2015**, *27*, 2653–2658, doi:10.1021/acs.chemmater.5b00481.
2. Feng, G. T.; Li, Y.Y; Colberts, F.J.M.; Lo, M.M.; Zhang, J.J.; Yang, F.; Jin, Y.Z.; Zhang, F.L.; Janssen, R.A.J.; Li, C.; Li, W.W. "Double-Cable" Conjugated Polymers with Linear Backbone toward High Quantum Efficiencies in Single-Component Polymer Solar Cells. *J Am Chem Soc* **2017**, *139*, 18647-18656, doi:10.1021/jacs.7b1049
3. Kyeong, M.K.; Lee, J.H.; Lee, K.H.; Hong, S.K. BODIPY-Based Conjugated Polymers for Use as Dopant-Free Hole Transporting Materials for Durable Perovskite Solar Cells: Selective Tuning of HOMO/LUMO Levels. *ACS Appl Mater & Interfaces* **2018**, 10 (27) , 23254-23262, doi:10.1021/acsami.8b05956
4. Zhao, J.; Zhao, S.L.; Xu, Z.; Qiao, B.; Huang, D.; Zhao, L.; Li, Y.; Zhu, Y.Q.; Wang P. Revealing the Effect of Additives with Different Solubility on the Morphology and the Donor Crystalline Structures of Organic Solar Cells. *ACS Appl Mater & Interfaces* **2016**, 8 (28) , 18231-1823, doi:10.1021/acsami.6b02671

5. Firdaus, Y.; Maffei, L.P.; Cruciani, F.; Muller, M.A.; Liu, S.J.; Lopatin, S.; Wehbe, N., Ndjawa, G.O.N.G.; Amassian, A.; Laquai, F.; Beaujuge P.M. Polymer Main-chain Substitution Effects on the Efficiency of Nonfullerence BHJ Solar Cells. *Adv Energy Mater* **2017**, 7, 1700834, doi:10.1002/aenm.20170083
6. Xia, D.D.; Wu, Y.; Wang, Q.; zhang, A.,D.; Li, C.; Lin, Y.Z.; Colberts, F.J.M.; van Franeker, J.J.; Janssen R.A.J.; Zhan X.W.; Hu, W.P.; Tang, Z.; Ma, W., Li, W.W. Effect of Alkyl Side Chains of Conjugated Polymer Donors on the Device Performance of Non-Fullerene Solar Cells. *Macromolecules* **2016**, 49, 6445–6454, doi: 10.1021/acs.macromol.6b01326
7. Kong, R.; Xiao, Z.; Xie, F.Y.; Jiang J.X.; Ding, L.M. A D–A copolymer donor containing an alkylthio-substituted thieno[3,2-b]thiophene unit. *New J Chem* **2017**, 41, 2895–2898, doi: 10.1039/c6nj03991j
8. An, Y.K.; Liao, X.F.; Cjem, L.; Yi, J.P.; Ai, Q.Y.; Xie, Q.; Huang, B.; Liu, F.; Jen, A.K.Y.; Chen, Y.W. Nonhalogen Solvent-Processed Asymmetric Wide-Bandgap Polymers for Nonfullerene Organic Solar Cells with Over 10% Efficiency. *Adv Func. Mater* **2018**, 28, 1706517.
9. Liao, X.F.; Yao, Z.Y.; Gao, K.; Shi, X.L.; Zuo, L.J.; Zhu, Z.L.; Chen, L.; Liu, F.; Chen, Y.W.; Jen, A.K.Y. Mapping Nonfullerene Acceptors with a Novel Wide Bandgap Polymer for High Performance Polymer Solar Cells. *Adv Energy Mater* **2018**, 8, 1801214, doi: 10.1002/aenm.201801214
10. Hao, D.; Li, M.; Liu, Y.H.; Li, C.H.; Bo, Z.S. Bis(carboxylate) substituted benzodithiophene based wide-bandgap polymers for high performance nonfullerene polymer solar cells. *Dyes Pigments* **2019**, 162, 120–125, doi: 10.1016/j.dyepig.2018.09.079
11. Li, G.D.; Xu, Q.Q.; Chang, C.M.; Fan Q.P.; Zhu, X.Q., Li, W.B., Guo, X., Zhang, M.J.; Wong, W.Y. High-Performance Nonfullerene Polymer Solar Cells Based on a Wide-Bandgap Polymer without Extra Treatment. *Macromol Rapid Commun* **2018**, 39, 1800660, doi: 10.1002/marc.201800660
